# Supplementary material for: Cost and cost effectiveness of reactive case detection (RACD), reactive focal mass drug administration (rfMDA) and reactive focal vector control (RAVC) to reduce malaria in the low endemic setting of Namibia: an analysis alongside a 2×2 factorial design cluster randomised controlled trial
Source: BMJ Open. 2022 Jun 23;12(6):e049050. doi: 10.1136/bmjopen-2021-049050 (PMC9226870; doi:10.1136/bmjopen-2021-049050)
Supplement: Supplementary data [file bmjopen-2021-049050supp001.pdf]

Supplement to:

Cost and cost-effectiveness of reactive case detection (RACD), reactive focal mass drug administration (rfMDA), and reactive focal vector control (RAVC) to reduce malaria in the low endemic setting of Namibia: an analysis alongside a 2x2 factorial design cluster randomized controlled trial

Table of Contents

Appendix 1a. Cumulative incidence of locally acquired malaria 2
Appendix 1b. Prevalence of quantitative PCR-detected infection 3
Appendix 2. Comparison groups 4
Appendix 3. Namibia Government salary scale 5

Appendix 1a. Cumulative incidence of locally acquired malaria

	No. of EAs	Incidence per 1000 person-years (95% CI)	p value	Incidence rate ratio (95% CI)	p value	Adjusted incidence rate ratio (95% CI)	p value
**Human reservoir**							
RACD	27	38.3 (23.0–53.6)	0.26	1 (reference)	0.51	1 (reference)	0.009
rfMDA	28	30.8 (12.8–48.7)		0.82 (0.26–1.37)		0.52 (0.16–0.88)	
**Mosquito reservoir**							
No RAVC	27	38.9 (20.7–57.1)	0.23	1 (reference)	0.41	1 (reference)	0.002
RAVC	28	30.2 (15.0–45.5)		0.78 (0.26–1.30)		0.48 (0.16–0.80)	
**Mosquito and human reservoir**							
RACD only	13	41.4 (21.5–61.2)	0.11	1 (reference)	0.32	1 (reference)	0.006
rfMDA+RAVC	14	25.0 (5.2–44.7)		0.62 (0.24–1.59)		0.26 (0.10–0.68)	

RACD: reactive case detection, rfMDA: reactive focal mass drug administration, RAVC: reactive vector control

Appendix 1b. Prevalence of quantitative PCR-detected infection

	N	Prevalence (95% CI)	p value	Prevalence ratio (95% CI)	p value	Adjusted prevalence ratio (95% CI)	p value
**Human reservoir**							
RACD	2150	3.78% (2.85-5.00)	0.46	1 (reference)	0.92	1 (reference)	0.009
rfMDA	1932	3.16% (2.14-4.65)		1.05 (0.03-2.07)		0.59 (0.21-0.98)	
**Mosquito reservoir**							
No RAVC	2030	4.07% (2.92-5.64)	0.15	1 (reference)	0.13	1 (reference)	0.002
RAVC	2052	2.92% (2.13-3.99)		0.61 (0.10-1.12)		0.36 (0.13-0.59)	
**Human and mosquito reservoir**							
RACD only	1016	3.70% (2.39-5.69)	0.04	1 (reference)	0.17	1 (reference)	0.004
rfMDA+RAVC	918	1.75% (0.99-3.09)		0.52 (0.20-1.32)		0.16 (0.05-0.55)	

RACD: reactive case detection, rfMDA: reactive focal mass drug administration, RAVC: reactive vector control

Appendix 2. Comparison groups

**rfMDA *versus* RACD**			Human reservoir	
			**RACD (28 clusters)**	**rfMDA (28 clusters)**
	Mosquito reservoir	**No RAVC**	RACD only	rfMDA only
		**RAVC**	RACD + RAVC	rfMDA + RAVC

**RAVC *versus* No RAVC**			Human reservoir	
			**RACD**	**rfMDA**
	Mosquito reservoir	**No RAVC (28 clusters)**	RACD only	rfMDA only
		**RAVC (28 clusters)**	RACD + RAVC	rfMDA + RAVC

**rfMDA +RAVC *versus* RACD only**			Human reservoir	
			**RACD**	**rfMDA**
	Mosquito reservoir	**No RAVC**	RACD only **(14 clusters)**	rfMDA only
		**RAVC**	RACD + RAVC	rfMDA + RAVC **(14 clusters)**

RACD: Reactive case detection, RAVC: Reactive vector control
rfMDA: Reactive focal Mass Drug administration

Appendix 3. Namibia Government salary scale

Grade	Low per annum		High per annum	
15	NAD	35,777	NAD	46,521
14	NAD	54,682	NAD	71,105
13	NAD	72,556	NAD	94,349
12	NAD	99,633	NAD	119,501
11	NAD	122,965	NAD	147,485
10	NAD	147,485	NAD	176,895
9	NAD	180,505	NAD	216,499
8	NAD	220,828	NAD	263,911
7	NAD	269,189	NAD	321,707
6	NAD	328,139	NAD	392,158
5	NAD	400,001	NAD	478,220
4	NAD	478,220	NAD	502,753
3	NAD	512,809	NAD	544,196
2	NAD	555,080	NAD	589,055
1	NAD	600,837	NAD	637,612
1C	NAD	625,110	NAD	663,373
1B	NAD	650,365	NAD	690,172
1A	NAD	663,373	NAD	703,975
