# Supplementary material for: Cost and cost effectiveness of reactive case detection (RACD), reactive focal mass drug administration (rfMDA) and reactive focal vector control (RAVC) to reduce malaria in the low endemic setting of Namibia: an analysis alongside a 2×2 factorial design cluster randomised controlled trial
Source: BMJ Open. 2022 Jun 23;12(6):e049050. doi: 10.1136/bmjopen-2021-049050 (PMC9226870; doi:10.1136/bmjopen-2021-049050)
Supplement: Author's manuscript [file bmjopen-2021-049050.draft_revisions.pdf]

BMJ Open is committed to open peer review. As part of this commitment we make the peer review history of every article we publish publicly available.

When an article is published we post the peer reviewers' comments and the authors' responses online. We also post the versions of the paper that were used during peer review. These are the versions that the peer review comments apply to.

The versions of the paper that follow are the versions that were submitted during the peer review process. They are not the versions of record or the final published versions. They should not be cited or distributed as the published version of this manuscript.

BMJ Open is an open access journal and the full, final, typeset and author-corrected version of record of the manuscript is available on our site with no access controls, subscription charges or pay-per-view fees (http://bmjopen.bmj.com).

If you have any questions on BMJ Open's open peer review process please email
info.bmjopen@bmj.com

BMJ Open

Cost and cost-effectiveness of reactive case detection (RACD), reactive focal drug mass administration (rfMDA), and reactive focal vector control (RAVC) to reduce malaria in the low endemic setting of Namibia: an analysis alongside a 2x2 factorial design cluster randomised controlled trial

Journal:	*BMJ Open*
Manuscript ID	bmjopen-2021-049050
Article Type:	Original research
Date Submitted by the Author:	26-Jan-2021

Complete List of Authors:	Ntuku, Henry; University of California San Francisco, Global Health Group, Malaria Elimination Initiative<br>Smith-Gueye, Cara; University of California San Francisco, Malaria Elimination Initiative<br>Scott, Valerie; University of California San Francisco, Malaria Elimination Initiative<br>Njau, Joseph; JoDon Consulting Group, LLC<br>Whittemore, Brooke; The University of Texas Southwestern Medical Center, Department of Pediatrics<br>Zelman, Brittany; University of California San Francisco, Malaria Elimination Initiative<br>Fox, Katie; University of California San Francisco, Malaria Elimination Initiative<br>Tambo, Munyaradzi; University of Namibia, Multidisciplinary Research Centre<br>Prach, Lisa; University of California San Francisco, Malaria Elimination Initiative<br>Wu, Lindsey; London School of Hygiene & Tropical Medicine, Department of Infection Biology<br>Schrubbe, Leah; University of California San Francisco, Malaria Elimination Initiative<br>Kang Dufour, Mi-Suk; University of California San Francisco, Division of Prevention Science<br>Mwilima, Agnes; Zambezi Ministry of Health and Social Services<br>Uusiku, Petrina; Namibia Ministry of Health and Social Services, National Vector-Borne Diseases Control Program<br>Sturrock, Hugh; University of California San Francisco, Malaria Elimination Initiative<br>Bennett, Adam; University of California San Francisco, Malaria Elimination Initiative, Global Health Group<br>Smith, Jennifer; University of California San Francisco, Malaria Elimination Initiative<br>Kleinschmidt, Immo; University of the Witwatersrand, School of Pathology, Faculty of Health Sciences; London School of Hygiene & Tropical Medicine, Department of Infectious Disease Epidemiology

	Mumbengegwi, Davis; University of Namibia, Multidisciplinary Research Centre<br>Gosling, Roly; University of California San Francisco, Global Health Group<br>Hsiang, Michelle; University of California San Francisco, Malaria Elimination Initiative; The University of Texas Southwestern Medical Center, Department of Pediatrics
Keywords:	Health economics < HEALTH SERVICES ADMINISTRATION & MANAGEMENT, Public health < INFECTIOUS DISEASES, INFECTIOUS DISEASES

SCHOLARONE™
Manuscripts

I, the Submitting Author has the right to grant and does grant on behalf of all authors of the Work (as defined in the below author licence), an exclusive licence and/or a non-exclusive licence for contributions from authors who are: i) UK Crown employees; ii) where BMJ has agreed a CC-BY licence shall apply, and/or iii) in accordance with the terms applicable for US Federal Government officers or employees acting as part of their official duties; on a worldwide, perpetual, irrevocable, royalty-free basis to BMJ Publishing Group Ltd ("BMJ") its licensees and where the relevant Journal is co-owned by BMJ to the co-owners of the Journal, to publish the Work in this journal and any other BMJ products and to exploit all rights, as set out in our licence.

The Submitting Author accepts and understands that any supply made under these terms is made by BMJ to the Submitting Author unless you are acting as an employee on behalf of your employer or a postgraduate student of an affiliated institution which is paying any applicable article publishing charge ("APC") for Open Access articles. Where the Submitting Author wishes to make the Work available on an Open Access basis (and intends to pay the relevant APC), the terms of reuse of such Open Access shall be governed by a Creative Commons licence – details of these licences and which Creative Commons licence will apply to this Work are set out in our licence referred to above.

Other than as permitted in any relevant BMJ Author's Self Archiving Policies, I confirm this Work has not been accepted for publication elsewhere, is not being considered for publication elsewhere and does not duplicate material already published. I confirm all authors consent to publication of this Work and authorise the granting of this licence.

For peer review only

Cost and cost-effectiveness of reactive case detection (RACD), reactive focal drug mass administration (rfMDA), and reactive focal vector control (RAVC) to reduce malaria in the low endemic setting of Namibia: an analysis alongside a 2x2 factorial design cluster randomised controlled trial

Henry Ntuku, PhD[1], Cara Smith Gueye, PhD[1], Valerie Scott, MPH[1], Joseph Njau, PhD[2], Brooke Whittemore, MPH[3], Brittany Zelman, MPH[1], Katie Fox, MSc[1], Munyaradzi Tambo, PhD[4], Lisa M. Prach, PhD[1], Lindsey Wu, PhD[5], Leah Schrubbe, MSc[1], Mi-Suk Kang Dufour, PhD[6], Agnes Mwilima, BA[7], Petrina Uusiku, MD[8], Hugh Sturrock, PhD[1], Adam Bennett, PhD[1], Jennifer L. Smith, PhD[1], Prof Immo Kleinschmidt, PhD[9,10,12], Prof Davis Mumbengegwi, PhD[4], Prof Roly Gosling, PhD[1], Michelle S. Hsiang, MD[1,3, 11]

[1]Malaria Elimination Initiative, Global Health Group, University of California San Francisco (UCSF), USA; [2]JoDon Consulting Group, LLC; Atlanta, GA , USA; [3]Department of Pediatrics, University of Texas Southwestern Medical Center, Dallas, TX, USA; [4]Multidisciplinary Research Centre, University of Namibia, Windhoek, Namibia; [5]Department of Infection Biology, London School of Hygiene and Tropical Medicine, [6]Division of Prevention Science, UCSF, USA; [7]Zambezi Ministry of Health and Social Services, Katima, Namibia; [8]National Vector-Borne Diseases Control Program, Ministry of Health and Social Services, Windhoek, Namibia; [9]School of Pathology, Faculty of Health Sciences, University of the Witwatersrand, Johannesburg, South Africa; [10]Department of Infectious Disease Epidemiology, London School of Hygiene and Tropical Medicine, London, UK; [11]Department of Pediatrics, UCSF, USA; [12]Southern African Development Community Malaria Elimination Eight Secretariat, Windhoek, Namibia.

*Correspondence to: Henry Ntuku MD PhD, Malaria Elimination Initiative, Global Health Group, University of California San Francisco (UCSF), 550 16th Street, 3rd Floor San Francisco, CA 94158, USA.

hntuku@path.org

Word count: 4153

For peer review only - http://bmjopen.bmj.com/site/about/guidelines.xhtml

ABSTRACT

Objectives

To estimate the cost and cost-effectiveness of reactive case detection (RACD), reactive focal drug mass administration (rfMDA), and reactive focal vector control (RAVC) to reduce malaria in a low endemic setting.

Setting

The study was part of a 2x2 factorial design cluster randomised controlled trial within the catchment area of 11 primary health facilities in Namibia.

Participants

Cost and outcome data were collected from the trial, which included 8948 community members that received interventions due to their residence within 500 meters of malaria index cases.

Outcome measures

The primary outcome was incremental cost effectiveness ratio (ICER) per in incident case averted. ICER per prevalent case, and per disability adjusted life years (DALY) averted were secondary outcomes, as were per unit interventions costs (USD) and personnel time. Outcomes were compared as: 1) rfMDA *versus* RACD, 2) RAVC *versus* No RAVC and 3) rfMDA+RAVC *versus* RACD only.

Results

rfMDA cost 1.1x more than RACD, and RAVC cost 1.7x more than no RAVC. Relative to RACD only, rfMDA+RAVC cost was double ($3,082 *versus* $1,553 per event). The ICERs for rfMDA *versus* RACD, RAVC *versus* No RAVC, and rfMDA+RAVC *versus* RACD only were $114, $1,472, and $842, per incident case averted, respectively. Using prevalent infections and DALYs as outcomes, trends were similar. Personnel was the main cost driver. The median personnel time to implement rfMDA was 20% lower than for RACD (30 *versus* 38 minutes per person), and per structure sprayed for RAVC, it was 34 minutes.

Conclusion

Implemented alone or in combination, rfMDA and RAVC were cost-effective in reducing malaria incidence and prevalence despite higher implementation costs in the intervention compared to control arms. Compared to RACD, rfMDA was time-saving. Cost and time requirements for the combined intervention could be decreased by implementing rfMDA and RAVC simultaneously by a single team.

Keywords: malaria, malaria elimination, Plasmodium falciparum, cost-effectiveness, economic, reactive, active case detection, mass drug administration, focal, indoor residual spraying, vector control, Anopheles

For peer review only - http://bmjopen.bmj.com/site/about/guidelines.xhtml

Strengths and limitations of this study:

- This is the first study to evaluate the costs and cost effectiveness of two new malaria transmission reducing strategies: reactive and focal mass drug administration and reactive vector control alone and in combination with reactive vector control.
- Estimates are derived from prospective data collection of costs and robust measures of effectiveness provided by a rigorous community randomised controlled trial
- The study design did not allow direct comparison between actual single interventions, since half of the clusters in each study arm compared received another intervention.
- In a secondary analysis, estimates of disability-adjusted life-years (DALYs) are derived from a study conducted in a different country

For peer review only

INTRODUCTION

In recent years, progress in malaria control and elimination, using standard approaches, has faltered [1], [2]. New malaria elimination approaches are needed, yet there are limited data on their costs and cost-effectiveness. In a context of declining donor funding, insufficient human resources and competing health priorities, economic evidence regarding malaria elimination interventions is needed to guide decision-making.

In areas with declining transmission intensity, a large proportion of infections are asymptomatic and they tend to cluster geographically and temporally [3]–[5]. Since malaria vectors are still present, these infections may seed further transmission in their immediate neighborhood, leading to focal outbreaks or clusters of malaria cases [6]. To prevent wider epidemics, community-based targeted interventions in response to passively identified malaria cases are recommended and implemented by programs aiming to interrupt transmission [7], [8]. One widely implemented intervention is reactive case detection (RACD), wherein individuals residing near passively detected index cases within a pre-defined radius are screened with a malaria rapid diagnostic test (RDT) and treated if results are positive.

For peer review only - http://bmjopen.bmj.com/site/about/guidelines.xhtml

However, RACD may have limited impact because RDTs fail to identify low density parasite infections, which predominate in low transmission settings [4], [9]–[11]. Circumventing this challenge of missed low-density infections reactive focal mass drug administration (rfMDA) is an alternative approach whereby the same household members and neighbors of index cases are presumptively treated with an antimalarial drug irrespective of infection status[12]. Reactive vector control (RAVC) is another promising elimination strategy, where focal indoor residual spraying (IRS) is targeted to households near index cases identified through passive case detection[12]. RAVC ensures sufficient IRS coverage in the areas most at risk of malaria, areas potentially insufficiently covered during a national IRS campaign, typically held months earlier before the malaria season has started. The combination of rfMDA and RAVC has been shown to be more effective than the interventions administered alone[12].

In addition to an intervention's effectiveness, costs and cost-effectiveness are key considerations in the decision-making process for National Malaria Control Programs and countries embarking on malaria elimination. Given that malaria control budgets are limited in elimination contexts, interventions or their combinations must be chosen based on expected cost and impact. While a few studies have examined costs associated with RACD [13], [14], there is very little information available on economic evaluations of rfMDA, and the limited data available report on the costs of focal mass drug administration following a community-wide screening[15] or mass drug administration (MDA), which is typically administered as a mass campaign [16]. To our knowledge there are no published economic data on RAVC or the combination of rfMDA and RAVC.

This paper reports the findings of an economic evaluation of three reactive strategies – RACD, rfMDA and RAVC – carried out as part of a cluster randomised controlled trial (CRCT) conducted in Zambezi Region, Namibia in 2017 [12]. The goal of the trial was to investigate the effectiveness and feasibility of rfMDA *versus* RACD both with and without RAVC as a community level intervention in

For peer review only - http://bmjopen.bmj.com/site/about/guidelines.xhtml

response to confirmed, passively identified malaria cases. Personnel time required for the different reactive focal interventions were also compared.

METHODS

Study site and description of the trial

The study was conducted from January to November 2017 in Zambezi region, northern Namibia. Since 2010, annual parasite incidence has been low (<15/1000 population), likely due to an increase in malaria funding and scaling up of effective interventions including improved case management, widespread use of RDTs, wide-scale implementation of preseason IRS campaigns with dichlorodiphenyltrichloroethane (DDT) or deltamethrin [17]. However, outbreaks have occurred in recent years, with an incidence of 33/1,000 population in 2016. Transmission is almost entirely due to *Plasmodium falciparum*. *Anopheles arabiensis* is the dominant vector, with the high transmission season occurring from January to June.

As reported elsewhere, the parent study was a CRCT with 2x2 factorial design [12][18]. In brief, 56 census enumeration areas (EAs) within the catchment area of 11 health facilities were randomised to receive rfMDA or RACD, and RAVC or no RAVC, resulting in four study arms: 1) RACD only, 2) RACD + RAVC, 3) rfMDA only, 4) rfMDA+RAVC (Table 1). The primary outcome was cumulative incidence of local malaria cases and the secondary outcome was infection prevalence as assessed in an endline survey. The total study population was 18,803. Study interventions were triggered by an index case diagnosed at the health facilities by either RDT or microscopy and reported through an electronic-based rapid reporting system. Within approximately two weeks of reporting, a study team consisting of a field investigator, nurse, and driver/data collector visited the index case household and neighboring households within a 500-meter radius with a target of enrolling at least 25 individuals for RACD and rfMDA. For RAVC, a separate team consisting of a spray team leader/driver and a spray assistant visited

For peer review only - http://bmjopen.bmj.com/site/about/guidelines.xhtml

the same location and sprayed the index case household and seven closest households. For each intervention, one follow-up visit was made if the enrolment target was not reached at the initial visit.

RACD consisted of testing for malaria using an RDT (CareStart Malaria HRP2/pLDH Pf/PAN, Access Bio, Somerset, New Jersey, USA) and treating those with positive results with artemether-lumefantrine (AL) (Coartem®, Novartis, Switzerland) and single low dose primaquine (Primaquine, Remedica, Cyprus) per national policy. Dried blood spots (DBS) were collected for molecular analysis. For rfMDA, eligible individuals were offered a presumptive, weight-appropriate, 3-day treatment course of AL only (Coartem®, Novartis, Switzerland). RAVC consisted of spraying ceilings and walls of sleeping structures with pirimiphos-methyl (Actellic 300 CS, Syngenta AG, Basel, Switzerland) per standard procedures [19]. To assess medication adherence, the team performed a follow-up pill count 7–10 days after the intervention for all RACD participants who were prescribed AL and among a sample of rfMDA participants.

The results of the trial showed that rfMDA and RAVC implemented alone and in combination reduced malaria incidence and parasite prevalence. Specifically, rfMDA and RAVC each reduced malaria incidence by nearly 50% when compared with their respective controls RACD and No RAVC, and rfMDA+RAVC reduced malaria incidence by 75% compared to RACD only. rfMDA and RAVC reduced prevalence by 41% and 64%, compared to RACD and No RAVC, respectively. rfMDA+RAVC reduced prevalence by 84% compared to RACD only [12].

Collection of costing data

The costing analysis was undertaken from the provider or program perspective. An ingredients-based or micro-costing approach was used for line item cost estimation. Inputs were identified, quantified, valued and classified into activity category [20]. Costs data were collected retrospectively from the financial expenditure records. No adjustments for inflation were made. Costs of items were valued according to

For peer review only - http://bmjopen.bmj.com/site/about/guidelines.xhtml

their market values in the year when they were purchased, in either Namibia dollars (NAD) or U.S. dollars (USD). Costs in NAD were converted to USD using the official 2017 average exchange rate of 1 USD per 14.28 NAD [21]. These data were entered into a costing tool in Microsoft Excel 2010.

Costs were classified as either capital or recurrent, showing the difference between investment once-off costs versus those that represent the running costs of ongoing program implementation. Capital costs included vehicles, computers, tablets, printers, photocopiers, and office furniture. For capital items like vehicles, office furniture, and computers estimated to have life years beyond the study period, a discounting rate of 3% and accounting depreciation value were considered before apportioning one year value of the equipment to the costs of the study (estimated project evaluation duration)[22]. Recurrent costs were grouped into one of four categories: consumables, services, personnel, or training and meetings. Consumables included field supplies and commodities related to the study intervention (RDTs, drugs and insecticides). Services included fuel for vehicles, airtime for staff, data bundles for tablets, maintenance of equipment, and office rental. Personnel costs included salaries and fringe benefits at private pay scale for the project staff, all of whom were full time. A percentage of their time spent for each intervention was applied, based on observation (see below). Costs were assigned to either RACD, rfMDA, or RAVC. For items shared by different intervention types, a percentage of use was allocated based on trial implementation data. The costing assessment was limited to the basic intervention level to resemble programmatic costs as close as possible. Costs related to research (i.e. international collaboration, the endline survey or molecular analyses) were excluded.

Personnel time

To assess the average time spent to complete the different interventions, a time and motion approach was used [23]. A field coordinator observed field personnel participating in the event, and recorded time spent by each staff member performing specific tasks. The sum of time spent by each personnel on each task was used to compute the total average time to complete the event. Data on personnel time were

For peer review only - http://bmjopen.bmj.com/site/about/guidelines.xhtml

collected as part of supervisory visits for a sample of 10 events for each strategy (rfMDA, RACD, and RAVC). Observed activities included: preparation time in the office, driving time, and event start and end times at the village. Data regarding the number of people treated (rfMDA) or tested and treated if positive (RACD) or the total number of structures sprayed (RAVC) were obtained from the trial database.

Collection of outcome data

Data on population at risk by study arms (defined as the total population in the study arm during the study period), number of events conducted, number of households sprayed and the total population residing in the sprayed households, number of people tested or presumptively treated, number of RDT-detected infections during RACD, and malaria case incidence and infection prevalence data were retrieved from the trial database [12] (**Table 1 and Appendix 1**).

Data Analysis

The following three comparison groups were used to compare intervention to control arms (**Appendix 2**): 1) rfMDA *versus* RACD, 2) RAVC *versus* no RAVC and 3) rfMDA+RAVC *versus* RACD only. Costing data reflect the way that interventions were implemented in the 2x2 factorial design. Specifically, for the rfMDA *versus* RACD comparison, expenses for the rfMDA group included those from the rfMDA only and rfMDA+RAVC arms, and expenses for the RACD group included those from the RACD only and RACD+RAVC arms. The same approach was used for the RAVC *versus* No RAVC comparison, where all expenses occurring in the RAVC arm (RACD+RAVC and rfMDA+RAVC) were compared to costs occurring in the No RAVC arm (RACD only and rfMDA only). With this approach, the No RAVC arm had non-zero costs and could be compared to RAVC. For the rfMDA+RAVC *versus* RACD only, the total costs of the combined interventions were compared to the total costs of RACD only.

To compare the costs, the following cost outcomes indicators were calculated: 1) total cost of the intervention; 2) cost per intervention event, calculated as the total cost of the strategy divided by

For peer review only - http://bmjopen.bmj.com/site/about/guidelines.xhtml

the total number of responses conducted; 3) cost per individual, calculated as the total cost of the strategy divided by either the total number of persons tested (for RACD), the total number of persons treated (for rfMDA), or the total number of persons residing in households sprayed (for RAVC); 4) cost per population at risk (PAR) calculated as the total cost of the intervention divided by the total population in the study arm in which the specific intervention was implemented.

For the cost of rfMDA and RAVC individually, we report relative costs to the control group because half of the clusters in these groups received a different intervention than the one being evaluated (i.e half of the EAs in the rfMDA group received RAVC, same for the RAVC group where half of the EAs received RACD and the other half received rfMDA) resulting in challenges to assess the cost and effectiveness of actual individual interventions. For the cost of rfMDA+RAVC combined, we report exact figures because the analyses are restricted to EAs receiving these specific interventions (**Appendix 2**).

For cost effectiveness, the incremental cost effectiveness ratio (ICER) was calculated as the difference in costs between intervention and control divided by the difference in their effect: *ICER= [(Cost of intervention) - (Cost of control)]/ [Effect intervention – Effect control]*. For the effect, we used three different impact outcomes measures; incidence rates (ICER per incident case averted), infection prevalence (ICER per prevalent infection averted), and disability adjusted life years DALYs (ICER per DALY averted). Models used to estimate adjusted incidence rate ratios and adjusted prevalence ratios with 95% confidence intervals (CIs), as reported in the main trial results paper [12], were used to predict incidence or infection prevalence. Using the population of the study area, the number of incident cases and prevalent infections was then estimated and used to calculate ICERs. DALYs averted were estimated using a mean loss of 1.18 DALYs per malaria case reported by a study conducted in neighboring Zimbabwe [24].

To define the interventions as cost effective, we used the Namibia's 2017 GDP per capita[25] and the World Health Organization economic evaluation guidelines on cost effectiveness thresholds for

For peer review only - http://bmjopen.bmj.com/site/about/guidelines.xhtml

low- and middle-income countries. The guidelines consider any program costs that are less than the national per capita gross domestic product (GDP) per DALY averted highly cost effective. Program costs that are less than three times the national per capita GDP per DALY averted are considered cost effective [26].

To enable comparisons between study arms, personnel time is presented as time to complete an intervention event (from arrival at index case household to departure from village of index case) excluding preparation time and driving time.

Sensitivity Analysis

A three-point estimation (PERT) sensitivity analysis was performed to validate the cost-effectiveness outcome results. Analysis focused on varying the effectiveness of the interventions by varying malaria incidence using interquartile range (IQR) of predicted incidence in interventions arms while keeping median estimates unchanged for control arms.

The discount rate, the costs of personnel, consumables and capital costs were also varied, and results obtained were compared to median estimates hereby referred to as best estimates. Capital costs including office space and furniture, were removed to estimate running costs for agencies that would use existing resources. For the commodity prices, a subsidized price of $15 per bottle of pirimiphos-methyl (Actellic 300 CS), as it can be obtained by the Ministry of Health and Social Services (MOHSS), was used. Finally, as malaria testing and treatment can be conducted by trained community health workers (CHW), their salaries (standard MOHSS daily rate of 260 NAD as daily labor costs), instead of those for study nurses, were used in the RACD versus rfMDA analysis.

Patient and Public involvement

Neither patients nor members of the public were involved in the design, or conduct, or reporting, or dissemination plans of the study.

For peer review only - http://bmjopen.bmj.com/site/about/guidelines.xhtml

RESULTS

During trial implementation, a total of 342 intervention events were conducted and 8 948 individuals received RACD or rfMDA, and RAVC or no RAVC (mean 26 participants/event). The total numbers of events conducted and individuals receiving each intervention by assigned group, and by comparison arm are shown in **Table 1.**

For the single intervention comparisons, the total cost of conducting rfMDA was 1.1x higher than RACD, while RAVC cost 1.7x more than no RAVC. For the combination intervention assessment, costs of rfMDA+RAVC were 1.8x higher than that for RACD only ($234,223 *versus* $127,312) (**Table 2**). By input category (**Figure 1**)**,** the main cost drivers for all three interventions were personnel costs (accounting for 66% of the RACD costs, 62% of rfMDA costs and 64% of RAVC costs) followed by capital costs, mainly vehicles (accounting for 12–13% of costs for any of the interventions). Consumables accounted for 9% of RACD costs, 14% of rfMDA costs and 12% of RAVC costs, with the main consumables being RDTs for RACD, antimalarial drugs for rfMDA and insecticide for RAVC.

Costing results by different comparison groups and units are shown in **Table 2** and **Figure 2.** For all groups, the cost per event, cost per individual and the cost per population at risk were higher in intervention groups compared to controls, and these costs were 1.1 to 1.2x more for rfMDA compared to RACD and 1.7 to 1.9x more for RAVC compared to no RAVC. When compared to RACD only, the combined intervention rfMDA+RAVC cost about twice more: $3,082 (*versus* $1,553), $113 (*versus* $58) and $54 (*versus* $27) per event, per individual and per population at risk respectively. In the RACD arms, there was a total of 114 infections identified, which translated to $3,112 per infection identified.

Using incidence and prevalence outcome data from the trial (**Appendices 1a and 1b**), the number of malaria incident cases and prevalent infections averted among study arms population were estimated, and ICERs were calculated for each comparison group to evaluate cost effectiveness. The base case scenario results for impact and cost effectiveness are shown in **Table 3**. Compared to the

For peer review only - http://bmjopen.bmj.com/site/about/guidelines.xhtml

standard of care RACD only, the combined intervention rfMDA+RAVC had an ICER of $842 per incident case averted and $1,812 per prevalent infection averted. The implementation of rfMDA resulted in an ICER of $114 per incident case averted and $162 per prevalent infection averted compared to RACD. RAVC was associated with an ICER of $1,472 per incident case averted and $2,670 per prevalent infection averted compared to no RAVC (**Figure 3**).

Using mean DALYs loss estimates [24] and the number of incident cases averted, the implementation of rfMDA and RAVC translated into an ICER of $97 per DALY averted for rfMDA compared to RACD, $1,248 per DALY averted for RAVC compared to no RAVC, and $714 per DALY averted for rfMDA+RAVC compared to RACD only (**Figure 3**). Given that Namibia's 2017 GDP per capita was $6,193 [25], both interventions as implemented in Zambezi region, singly or combined would be considered highly cost-effective health interventions.

Sensitivity analysis

The parameters included in the sensitivity analysis and the change in total costs and ICERs are detailed in **Table 4** and **Figure 4**. For all three comparison groups, the ICER per incident case averted was sensitive to variations in predicted malaria incidence. Changes in the other parameters (i.e. replacing nurses by CHW for malaria testing and treatment, using a discounted price of pirimiphos-methyl (Actellic 300 CS) and conducting the analysis on recurrent expenditures) reduced the overall costs of interventions with similar reductions on cost per event, cost per person, cost per population at risk, but did not have a significant impact on ICERs.

Personnel time

The time spent by teams to enroll a participant was longer for RACD compared to rfMDA with a median personnel-minutes per participant enrolled of 38 minutes (IQR: 35-44) versus 30 minutes (IQR: 25-38). The median personnel-minutes per structure sprayed for RAVC was 34 minutes (IQR: 29-38). With an

For peer review only - http://bmjopen.bmj.com/site/about/guidelines.xhtml

average of 4 people per household, the median personnel-minutes per individual protected with RAVC was 9 minutes. The median preparation time was 33 minutes for rfMDA and 32 minutes for RACD and RAVC. For all interventions, the teams spent on average 2 hours per event travelling to and from the index case household.

DISCUSSION

Results of this study show that compared to the standard of care, reactive focal interventions (rfMDA, RAVC and rfMDA+RAVC) used in a low transmission Sub Saharan African setting, are cost effective, when considering outcomes of malaria incidence or prevalence, or DALY averted. In addition, rfMDA was time saving compared to RACD. These results, taken together with the main trial findings on effectiveness of these interventions, suggest that in such settings, a change in policy and practice from RACD to rfMDA and/or RAVC should be strongly considered [11].

It is established that the investment needed to achieve malaria elimination will be higher per case than that of standard malaria control [27], [28]. Indeed the costs per person reported in this study are significantly higher than the ones reported by White *et al* in a systematic review on costs and cost-effectiveness of malaria control interventions ($2.2 for mosquito nets and $6.7 for IRS)[29]. However, in the elimination context, a comparison to the current standard of care, which often includes RACD, is more useful. Further, it is generally accepted that the long-term benefits of elimination outweigh the costs, making the pursuit worthwhile [27], [28].

Although rfMDA costs are marginally higher compared to RACD, the difference is more a reflection of economies of scale. Due to the way in which we have calculated costs in relation to events, we show a conservative assessment of costs and cost-effectiveness. For example, because RACD was less effective, more RACD events were carried out and more people tested, lowering the cost per

For peer review only - http://bmjopen.bmj.com/site/about/guidelines.xhtml

person. In the only other reported study of costs for RACD from Indonesia [30], the cost per individual screened, without major start-up capital costs, was lower ($28 compared to $39 in this study) but that study had more individuals enrolled per RACD event (42 individuals compared to 25 individuals per event in this study). Also, the total costs for the combination intervention and RACD only should not be compared to those of the individual interventions because the combination intervention was only conducted in half the study clusters.

Both rfMDA and RAVC implemented alone or in combination remained cost effective in with varying effectiveness. The sensitivity analysis also provided an indication on how the costs of interventions may be reduced in the future. First, the costs would be reduced by more than 10% if the interventions are to be implemented by MoHSS using existing resources (fuel, maintenance) (**Table 4**). Costs could be further reduced through better integration of staff. Consistent with other studies of malaria community level interventions [31], [32], personnel costs were the main cost driver. Conducting reactive interventions is challenging and resource intensive tasks, requiring dedicated teams working throughout the malaria transmission season to achieve high coverage of interventions. A CHW program could be considered as a potentially cost-effective approach for the delivery of health services to communities [33]. CHWs are used in similar settings to deliver reactive interventions[15], [34], [35] and Namibia currently has a pool of trained and accredited CHW to test and treat malaria who could be used in the implementation of reactive interventions. A community-based, self-administered treatment approach using village health workers is currently being tested in a randomised controlled trial in the Gambia as a cost effective way to deliver rfMDA [36]. Finally, considerable cost savings can be made by combining rfMDA or RACD activities with RAVC, which we did not do in the study due to protocol design.

Despite the time requirements associated with rfMDA, such as reviewing potential contraindications and adverse effects, and ensuring treatment compliance, the time required to implement rfMDA was less, due to the time it takes to conduct an RDT, wait for the results, share the

For peer review only - http://bmjopen.bmj.com/site/about/guidelines.xhtml

result with the participant, and then provide treatment if indicated. Not surprisingly, the median personnel-time per participant was shorter in the rfMDA arms compared to RACD. The shorter time per participant in rfMDA is expected to result in a higher number of individuals covered by rfMDA in a program implementation setting, potentially leading to a lower cost per person due to economies of scale.

There were some limitations to this study. The intervention was not designed to minimize costs and maximize cost effectiveness. For example, in rfMDA arms, more than the targeted number of 25 individuals could have been enrolled per event with the same staff working time and could have resulted in reduced costs per person enrolled. The study design did not allow direct comparison between actual single interventions, since half of the EAs in the RACD and the rfMDA comparison groups received RAVC, and for RAVC, half of the EAs received RACD and half received rfMDA. Although all efforts were made to exclude costs related to research activities, it was not possible to identify the costs related to some research activities such as, time needed to obtain informed consent, conduct interviews, and collect blood samples, and therefore, these costs could not be excluded from the analysis. Program implementation costs of these interventions are therefore likely to be lower. Estimates of DALYs used in this study are derived from a study conducted in a different country, but malaria morbidity and mortality are likely to be similar.

In conclusion, in the low transmission setting of Namibia, rfMDA and RAVC implemented as singly or in combination are highly cost-effective interventions that can substantially reduce malaria transmission. Compared to RACD, rfMDA was time-saving, and for the combination intervention, the additional cost and time requirements for RAVC could be minimized by having a single team implement both rfMDA and RAVC at the same time. Given the frequent occurrence of outbreaks in low transmission areas of southern Africa, applying the rfMDA +/- RAVC approach might be very effective at bringing outbreaks rapidly under control. These results offer a good indication of the best value for money,

For peer review only - http://bmjopen.bmj.com/site/about/guidelines.xhtml

additional research is needed to provide information on other factors to guide decision making such as affordability and budget impact. Findings are likely to be generalizable to other low transmission settings where cases of malaria or even other infectious diseases, are highly clustered around index cases, and therefore provide a framework for decision making by countries eliminating malaria and other infectious diseases.

For peer review only

For peer review only - http://bmjopen.bmj.com/site/about/guidelines.xhtml

Table 1. Main trial design and costing outcome measures

		Human reservoir	
		**RACD***<br>(reactive case detection)<br>- 28 clusters<br>- Pop at risk: 9898<br>- No of events: 178<br>- No of people: 4701<br>- No of RDT identified infections: 114	**rfMDA†**<br>(reactive focal mass drug administration)<br>- 28 clusters<br>- Pop at risk: 8905<br>- No of events: 164<br>- No of people: 4247
Mosquito reservoir	**No RAVC** (no reactive focal vector control)<br>- 28 clusters<br>- Pop at risk: 9339<br>- No of events: 170<br>- No of people: 4369	**RACD only arm**<br>- 14 clusters<br>- Pop at risk: 4742<br>- No of events: 82<br>- No of people: 2188<br>- No of RDT identified infections: 52	**rfMDA only arm**<br>- 14 clusters<br>- Pop at risk: 4597<br>- No of events: 88<br>- No of people: 2181
	**RAVC‡** (reactive focal vector control)<br>- 28 clusters<br>- Pop at risk: 9464<br>- No of events: 172<br>- No of people: 4032	**RACD + RAVC arm**<br>- 14 clusters<br>- Pop at risk: 5156<br>- No of events: 96<br>- No of people: 2513<br>- No of RDT identified infections: 62	**rfMDA + RAVC arm**<br>- 14 clusters<br>- Pop at risk: 4308<br>- No of events: 76<br>- No of people: 2066

*RACD: Reactive case detection, † rfMDA: Reactive focal Mass Drug administration, ‡RAVC: Reactive vector control

For peer review only - http://bmjopen.bmj.com/site/about/guidelines.xhtml

Table 2. Total and unit costs per comparison group (Cost in 2017 USD)

	Average costs (Start-up and recurrent)					Recurrent costs only				
Comparison groups	Total costs	Cost per PAR#	Cost per event	Cost per person	Cost per infection identified	Total costs	Cost per PAR	Cost per event	Cost per person	Cost per infection identified
Human reservoir										
RACD*	$354,750	$35.8	$1,993	$75.5	$3,112	$309,368	$31.3	$1,738	$65.8	$2,714
rfMDA†	$368,321	$41.4	$2,246	$86.7	-	$322,940	$36.3	$1,969	$76.1	-
Mosquito reservoir										
No RAVC‡	$261,409	$28.0	$1,537	$59.8	-	$227,837	$24.4	$1,340	$52.2	-
RAVC	$461,661	$48.8	$2,684	$114.5	-	$404,470	$42.7	$2,352	$100.3	-
Human and mosquito reservoir										
RACD only	$127,312	$26.9	$1,553	$58.2	$2,448	$110,526	$23.3	$1,348	$50.5	$2,126
rfMDA+RAVC	$234,223	$54.4	$3,082	$113.4	-	$205,628	$47.7	$2,706	$99.5	-

#PAR: population at risk, *RACD: Reactive case detection, † rfMDA: Reactive focal Mass Drug administration, ‡RAVC: Reactive vector control

For peer review only

Table 3. Impact indicators and incremental cost effectiveness ratio (ICER) for the three comparison groups (cost in 2017 USD)

Comparison groups	Predicted incidence per 1000 person-years (95% CI)	No. of Incident cases	No. of Incident cases averted	DALYs averted	Predicted infection prevalence (95% CI)	No. of Prevalent infections	No. of Prevalent infections averted	ICER per incident case averted	ICER per prevalent infection averted	ICER per DALY averted
Human reservoir										
RACD	42.1 (30.0-54.2)	396	Ref	Ref	3.3% (2.7-4.0)	310	Ref	Ref	Ref	Ref
rfMDA	29.5 (19.0-40.0)	277	119	140	2.4% (1.6-3.2)	226	84	$114	$162	$97
Mosquito reservoir										
No RAVC	43.1 (28.7-57.6)	405	Ref	Ref	3.5% (2.7-4.3)	329	Ref	Ref	Ref	Ref
RAVC	28.6 (21.7-35.4)	269	136	160	2.7% (1.8-3.3)	254	75	$1,472	$2,670	$1,248
Human and mosquito reservoir[#]										
RACD only	52.5 (30.4-74.6)	238	Ref	Ref	3.1% (2.0-4.1)	140	Ref	Ref	Ref	Ref
rfMDA+RAVC	24.6 (14.7-34.5)	111	127	150	1.8% (0.5-2.9)	81	59	$842	$1,812	$714

RACD: Reactive case detection, rfMDA: Reactive focal Mass Drug administration, RAVC: Reactive vector control, ICER: Incremental cost effectiveness ratio, DALY: disability-adjusted life year.

[#]Based on half of the clusters (14)

For peer review only - http://bmjopen.bmj.com/site/about/guidelines.xhtml

Table 4. Results of sensitivity analysis and scenario analyses (cost in 2017 USD)

		Results/Implications					
		rfMDA		RAVC		rfMDA+RAVC	
**Parameter**	**Base value/ Sensitivity analysis value(s)**	**Total costs**	**ICER (*vs* RACD) per incident case averted**	**Total costs**	**ICER (*vs* No RAVC) per incident case averted**	**Total costs**	**ICER (*vs* RACD only) per incident case averted**
Predicted incidence	Mean	-	$114	-	$1,472	-	$842
	Lower limit	-	$63	-	$996	-	$625
	Upper limit	-	$679	-	$2,781	-	$1,304
Discount rate	3%	$368,321	$114	$461,661	$1,472	$234,223	$842
	0%	$366,699	$109	$460,035	$1,464	$233,246	$840
	5%	$369,187	$116	$463,093	$1,477	$234,940	$851
Use of CHW	Nurses	$368,321	$114	-	-	$234,223	$842
	CHW for RACD and rfMDA	$313,715	$111	-	-	$206,920	$840
Capital costs	Included	$368,321	$114	$461,661	$1,472	$234,223	$842
	Costs treated as recurrent	$322,940	$114	$404,470	$1,299	$205,628	$749
Actellic cost	$38.80			$461,663	$1,472	$234,226	$842
	$15	-	-	$449,659	$1,381	$228,628	$799

For peer review only - http://bmjopen.bmj.com/site/about/guidelines.xhtml

RACD: reactive case detection, rfMDA: reactive focal mass drug administration, RAVC: reactive vector control, ICER: Incremental cost effectiveness ratio. Cost in 2017 USD

For peer review only

Figure legends

Figure 1. Cost category breakdown for RACD, rfMDA, RAVC

Figure 2. Start-up and recurrent costs by comparison groups for cost per event (2a), cost per person (2b), and cost per PAR (2c).

Figure 3. Incremental cost effectiveness ratio (ICER) of RACD vs rfMDA, No RAVC vs RAVC and RACD only vs rfMDA+RAVC per incident case averted, prevalent infection averted, and DALY averted.

Figure 4. Tornado diagram of change in the incremental cost effectiveness ratio per incident case averted for rfMDA *vs* RACD (4a), RAVC *vs* No RAVC (4b) and rfMDA+RAVC *vs* RACD only (4c). The values in brackets are the range over which the parameter was varied. The vertical line is the baseline value of the ICER per incident case averted. The blue bars show the direction and magnitude of change in the ICER, when the input variable is set to its lower value and the red bars show the direction and magnitude of change when the input variable is set to its higher value. For the nurses category, costs are reduced on both intervention and control arms, resulting in a marginal change in the ICER that is not visible on the graph.

For peer review only - http://bmjopen.bmj.com/site/about/guidelines.xhtml

Declarations

Ethical approval and consent to participate

The main trial received ethical approval from the Namibia Ministry of Health and Social Services, and the Institutional Review Boards of the University of Namibia, University of California San Francisco and London School of Medicine and Hygiene. Consent was not obtained from study staff being observed as it was considered part of study supervision.

Consent for publication

All authors have read and approved the final manuscript.

Data availability statement

Data supporting the conclusions of this article is available upon request.

Competing Interests

The authors have no competing interests to report.

Funding

This work was supported by grants from the Novartis Foundation (A122666), the Bill and Melinda Gates Foundation (OPP1138299), and Horchow Family Fund, (grant 5300375400 to MSH).

Authors' contributions

MSH, RG, and IK led the conception and study design. DM, JS, AB, HS, and PU contributed to the study design. HN, CSG, VS, BZ, MT, KF, LMP, LW, LS and AM supported data collection. HN and MSH performed data cleaning and data analyses. JN, BW and MSD supported data analysis. HN wrote the first draft of the manuscript. HN and MSH wrote the final draft of the manuscript. All authors read and approved the final manuscript.

For peer review only - http://bmjopen.bmj.com/site/about/guidelines.xhtml

Acknowledgements

The authors would like to express their gratitude to the national and regional Ministry of Health and Social Services, and the local traditional leadership for their support. The authors would also like to acknowledge Megan McElroy, Simataa Nyathi, Flavian Libita, and Chaze Sibeya for their support in the preparation and collection of field data.

For peer review only

References

1. Feachem RG, Phillips AA, Hwang J, et al. Shrinking the malaria map: progress and prospects. *Lancet*. 2010;376(9752):1566–1578. doi:10.1016/S0140-6736(10)61270-6: 10.1016/S0140-6736(10)61270-6

2. WHO. *World Malaria Report 2019*.; 2019.

3. Okell LC, Ghani AC, Lyons E, Drakeley CJ. Submicroscopic Infection in Plasmodium falciparum-Endemic Populations: A Systematic Review and Meta-Analysis. *J Infect Dis*. 2009;200(10):1509-1517. doi:10.1086/644781

4. Sturrock HJW, Hsiang MS, Cohen JM, et al. Targeting Asymptomatic Malaria Infections : Active Surveillance in Control and Elimination. *PLoS Med*. 2013;10(6):e1001467. doi:10.1371/journal.pmed.1001467

5. Björkman A, Cook J, Sturrock H, et al. Spatial Distribution of Falciparum Malaria Infections in Zanzibar : Implications for Focal Drug Administration Strategies Targeting Asymptomatic Parasite Carriers. *Clin Infect Dis*. 2017;64(9):1236-1243. doi:10.1093/cid/cix136

6. Bousema T, Okell L, Felger I, Drakeley C. Asymptomatic malaria infections: detectability, transmissibility and public health relevance. *Nat Rev Microbiol*. 2014;12(12):833-840.

7. Moonen B, Cohen JM, Tatem AJ, et al. A framework for assessing the feasibility of malaria elimination. *Malar J*. 2010;9(1):322. doi:10.1186/1475-2875-9-322

8. Cotter C, Sturrock HJW, Hsiang MS, et al. The changing epidemiology of malaria elimination : new strategies for new challenges. *Lancet*. 2013;382(9895):900-911. doi:10.1016/S0140-6736(13)60310-4

9. Stresman GH, Baidjoe AY, Stevenson J, et al. Focal Screening to Identify the Subpatent Parasite Reservoir in an Area of Low and Heterogeneous Transmission in the Kenya Highlands. *J Infect Dis*. 2015;212:1768-1777. doi:10.1093/infdis/jiv302

10. Cook J, Xu W, Msellem M, et al. Mass Screening and Treatment on the Basis of Results of a Plasmodium falciparum -Speci fi c Rapid Diagnostic Test Did Not Reduce Malaria Incidence in Zanzibar. *J Infect Dis*. 2015;211:1476-1483. doi:10.1093/infdis/jiu655

11. WHO. *Evidence Review Group Report on Mass Drug Administration , Mass Screening and Treatment and Focal Screening and Treatment for Malaria*.; 2015.

12. Hsiang MS, Ntuku H, Roberts KW, et al. Effectiveness of reactive focal mass drug administration and reactive focal vector control to reduce malaria transmission in the low malaria-endemic setting of Namibia: a cluster-randomised controlled, open-label, two-by-two factorial design trial. *Lancet*. 2020;395(10233):1361-1373. doi:10.1016/S0140-6736(20)30470-0

13. Zelman BW, Baral R, Zarlinda I, et al. Costs and cost - effectiveness of malaria reactive case detection using loop - mediated isothermal amplification compared to microscopy in the low transmission setting of Aceh Province , Indonesia. *Malar J*. Published online 2018:1-12. doi:10.1186/s12936-018-2361-y

14. Larson BA, Ngoma T, Silumbe K, et al. A framework for evaluating the costs of malaria elimination interventions : an application to reactive case detection in Southern Province of Zambia , 2014.

For peer review only - http://bmjopen.bmj.com/site/about/guidelines.xhtml

Malar J. 2016;15(408):1-15. doi:10.1186/s12936-016-1457-5

15. Yukich JO, Scott C, Silumbe K, et al. Cost-Effectiveness of Focal Mass Drug Administration and Mass Drug Administration with Dihydroartemisinin–Piperaquine for Malaria Prevention in Southern Province, Zambia: Results of a Community-Randomized Controlled Trial. *Am J Trop Med Hyg*. 2020;103(2_Suppl):46-53. doi:10.4269/ajtmh.19-0661

16. Kaneko A, Taleo G, Kalkoa M, Yamar S, Kobayakawa T, Björkman A. Malalria eradictaion on islands. *Lancet*. 2000;356.

17. Ministry of Health and Social Services. Namibia. *National Policy on Malaria*.; 2014.

18. Medzihradsky O, Kleinschmidt I, Mumbengegwi D, Roberts K, McCreesh P, Dufour K. Study protocol for a cluster randomised controlled factorial design trial to assess the effectiveness and feasibility of reactive focal mass drug administration and vector control to reduce malaria transmission in the low endemic setting of Namibia. *BMJ Open*. 2018;8(e019294).

19. World Health Organization. *Indoor Residual Spraying: An Operational Manual for Indoor Residual Spraying (IRS) for Malaria Transmission Control and Elimination.*

20. Drummond M, Sculper M, Tornace G, O'Brien B, Stoddart G. *Methods for the Economic Evaluation of Health Care Programmes*. 3rd ed. (Oxford, ed.). Oxford University Press; 2005.

21. OANDA. Currency converter. Accessed October 9, 2019. http://www.oanda.com/currency/historical-rates/

22. Walker D, Kumaranayake L. Allowing for differential timing in cost analyses : discounting and annualization. *Health Policy Plan*. 2002;17(1):112-118.

23. Lopetegui M, Yen P-Y, Lai A, Jeffries J, Embi P, Payne P. Time motion studies in healthcare: what are we talking about? *J Biomed Inform*. 2014;49:292-299. doi:10.1016/j.jbi.2014.02.017

24. Gunda R, Chimbari MJ, Mukaratirwa S. Assessment of Burden of Malaria in Gwanda District, Zimbabwe, Using the Disability Adjusted Life Years. *Int J Environ Res Public Health*. 2016;13(2):244. doi:10.3390/ijerph13020244

25. World Bank. International Comparison Program database. Published 2017. www.data.worldbank.org/indicator/

26. WHO. Cost effectiveness and strategic planning (WHO-CHOICE). http://www.who.int/choice/cost-effectiveness/en/

27. Sabot O, Cohen JM, Hsiang MS, et al. Costs and financial feasibility of malaria elimination. *Lancet*. 2010;376(9752):1604-1615. doi:10.1016/S0140-6736(10)61355-4

28. Shretta R, Avanceña AL V, Hatefi A. The economics of malaria control and elimination: a systematic review. *Malar J*. 2016;15(1):593. doi:10.1186/s12936-016-1635-5

29. White MT, Conteh L, Cibulskis R, Ghani AC. Costs and cost-effectiveness of malaria control interventions - a systematic review. *Malar J*. 2011;10(1):337. doi:10.1186/1475-2875-10-337

30. Zelman BW, Baral R, Zarlinda I, et al. Costs and cost ‑ effectiveness of malaria reactive case detection using loop ‑ mediated isothermal amplification compared to microscopy in the low transmission setting of Aceh Province , Indonesia. *Malar J*. Published online 2018:1-12.

For peer review only - http://bmjopen.bmj.com/site/about/guidelines.xhtml

doi:10.1186/s12936-018-2361-y

31. Silumbe K, Yukich JO, Hamainza B, et al. Costs and cost-effectiveness of a large-scale mass testing and treatment intervention for malaria in. *Malar J*. 2015;14(211):1-13. doi:10.1186/s12936-015-0722-3

32. Drake TL, Okello G, Njagi K, et al. Cost analysis of school-based intermittent screening and treatment of malaria in Kenya. *Malar J*. 2011;10(1):273. doi:10.1186/1475-2875-10-273

33. Vaughan K, Kok MC, Witter S, Dieleman M. Costs and cost-effectiveness of community health workers : evidence from a literature review. *Hum Resour Health*. 2015;13:71. doi:10.1186/s12960-015-0070-y

34. Liverani M, Nguon C, Sok R, et al. Improving access to health care amongst vulnerable populations: a qualitative study of village malaria workers in Kampot, Cambodia. *BMC Health Serv Res*. 2017;17(1):335. doi:10.1186/s12913-017-2282-4

35. Eisele TP, Bennett A, Silumbe K, et al. Impact of Four Rounds of Mass Drug Administration with Dihydroartemisinin–Piperaquine Implemented in Southern Province, Zambia. *Am J Trop Med Hyg*. 2020;103(2_Suppl):7-18. doi:https://doi.org/10.4269/ajtmh.19-0659

36. Okebe J, Ribera JM, Balen J, et al. Reactive community-based self- administered treatment against residual malaria transmission : study protocol for a randomized controlled trial. *Trials*. Published online 2018:1-8.

For peer review only

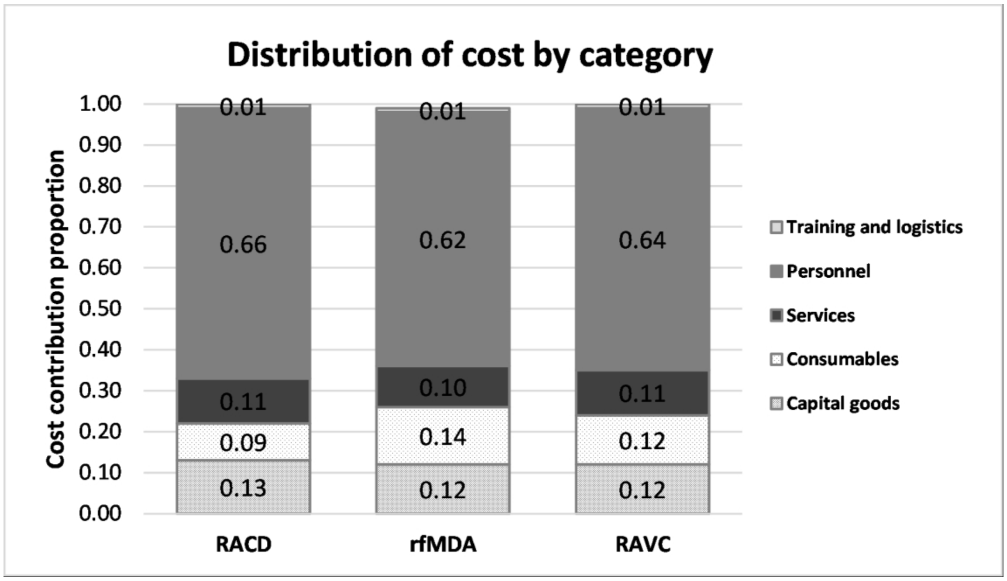

Figure 1. Cost category breakdown for RACD, rfMDA, RAVC

156x90mm (300 x 300 DPI)

a)

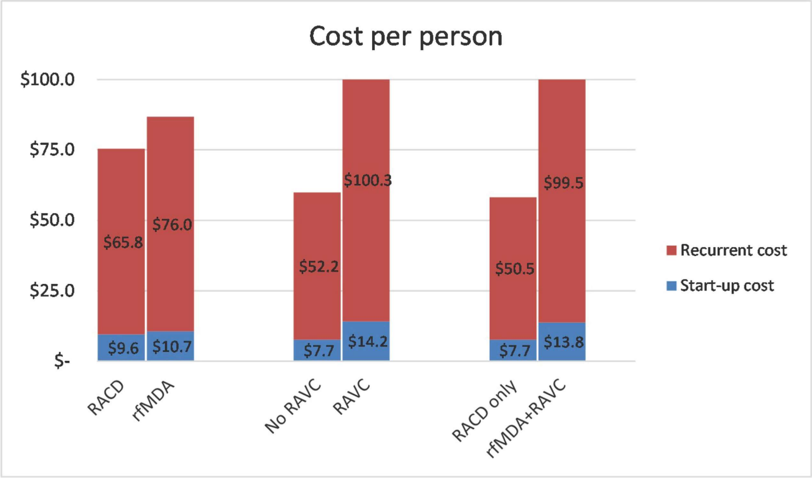
Cost per person
$100.0
$75.0
$50.0
$25.0
$-
$65.8
$76.0
$52.2
$100.3
$50.5
$99.5
$9.6
$10.7
$7.7
$14.2
$7.7
$13.8
RACD
rFMDA
No RAVC
RAVC
RACD only
rfMDA+RAVC
Recurrent cost
Start-up cost

b)

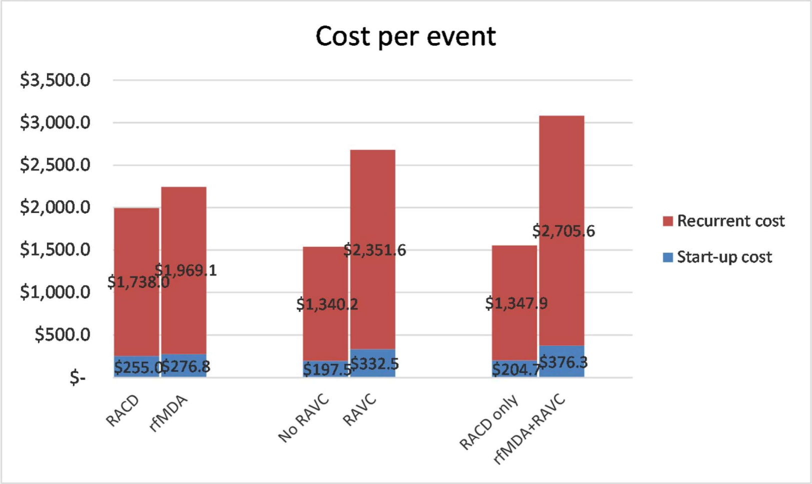
Cost per event
$3,500.0
$3,000.0
$2,500.0
$2,000.0
$1,500.0
$1,000.0
$500.0
$-
$1,738.0
$1,969.1
$1,340.2
$2,351.6
$1,347.9
$2,705.6
$255.0
$276.8
$197.5
$332.5
$204.7
$376.3
RACD
rfMDA
No RAVC
RAVC
RACD only
rfMDA+RAVC
Recurrent cost
Start-up cost

c)

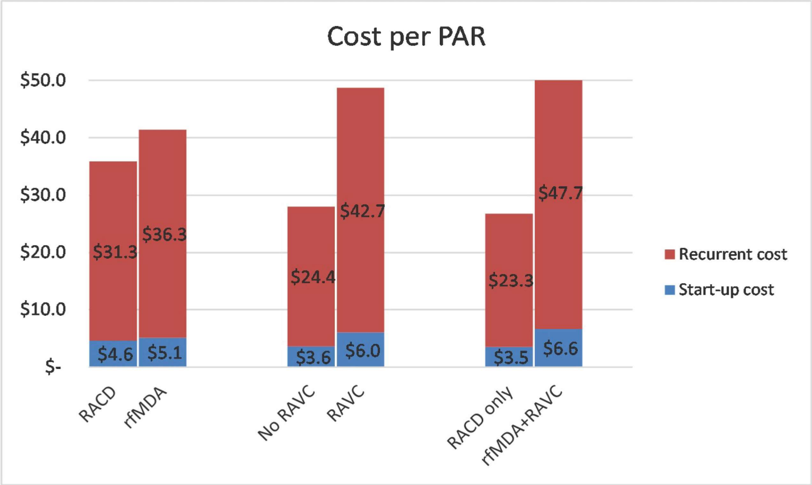
Cost per PAR
$50.0
$40.0
$30.0
$20.0
$10.0
$-
$31.3
$36.3
$24.4
$42.7
$23.3
$47.7
$4.6
$5.1
$3.6
$6.0
$3.5
$6.6
RACD
rfMDA
No RAVC
RAVC
RACD only
rfMDA+RAVC
Recurrent cost
Start-up cost

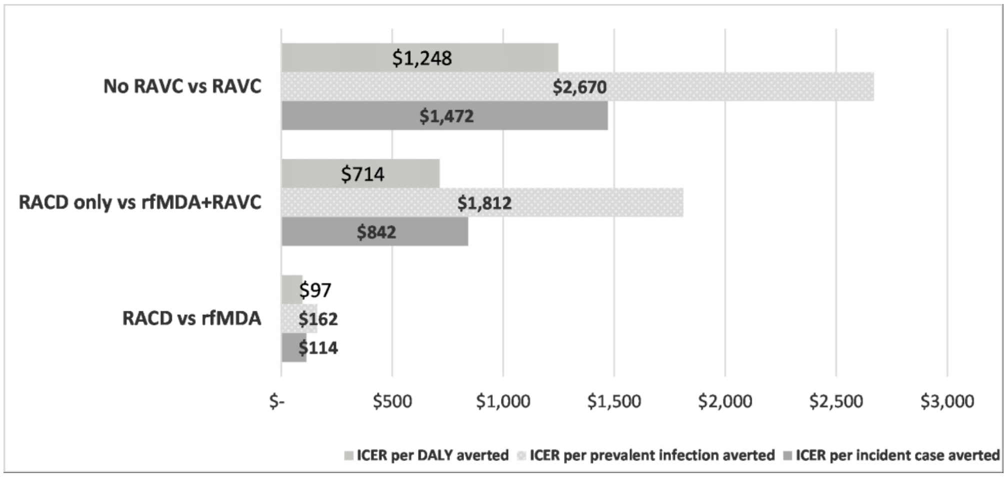

Figure 3. Incremental cost effectiveness ratio (ICER) of RACD vs rfMDA, No RAVC vs RAVC and RACD only vs rfMDA+RAVC per incident case averted, prevalent infection averted, and DALY averted.

191x90mm (300 x 300 DPI)

a)

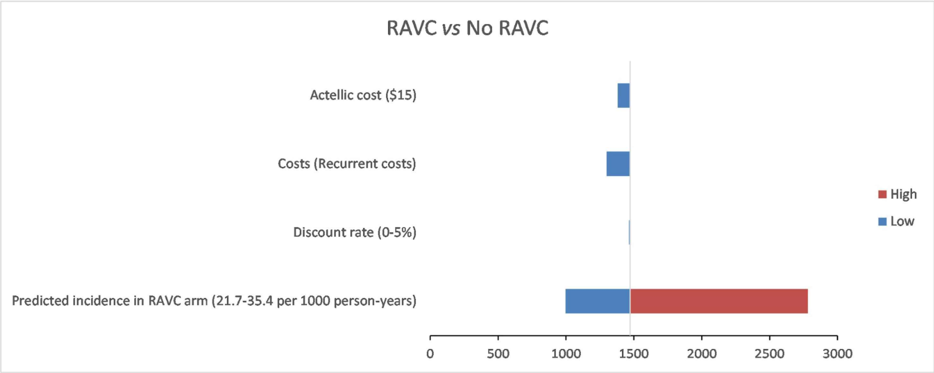
RAVC vs No RAVC
Actellic cost ($15)
Costs (Recurrent costs)
Discount rate (0-5%)
Predicted incidence in RAVC arm (21.7-35.4 per 1000 person-years)
High
Low
0
500
1000
1500
2000
2500
3000

b)

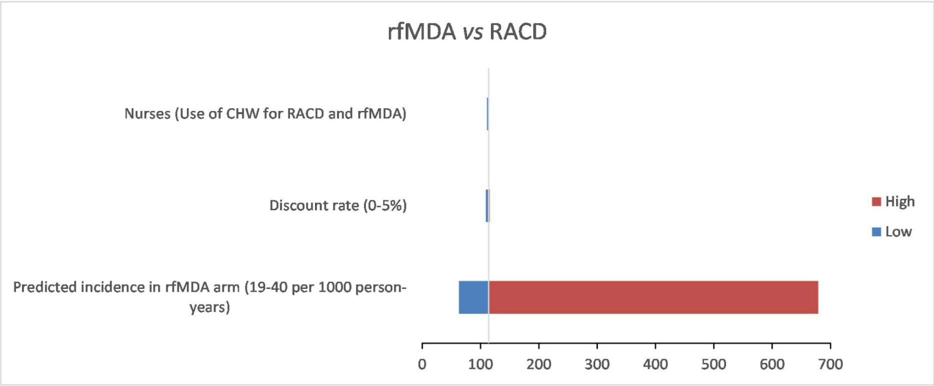
rfMDA vs RACD
Nurses (Use of CHW for RACD and rfMDA)
Discount rate (0-5%)
Predicted incidence in rfMDA arm (19-40 per 1000 person-years)
High
Low
0
100
200
300
400
500
600
700

c)

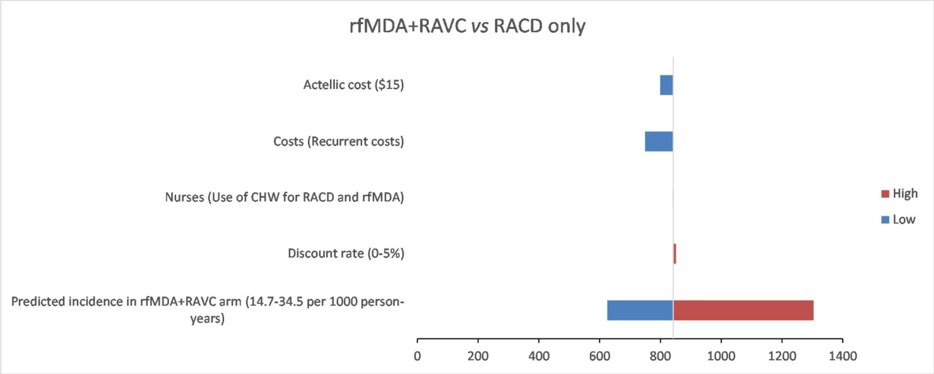
rfMDA+RAVC vs RACD only
Actellic cost ($15)
Costs (Recurrent costs)
Nurses (Use of CHW for RACD and rfMDA)
Discount rate (0-5%)
Predicted incidence in rfMDA+RAVC arm (14.7-34.5 per 1000 person-years)
High
Low
0
200
400
600
800
1000
1200
1400

Supplement to:

Cost and cost-effectiveness of reactive case detection (RACD), reactive focal drug mass administration (rfMDA), and reactive focal vector control (RAVC) to reduce malaria in the low endemic setting of Namibia: an analysis alongside a 2x2 factorial design cluster randomized controlled trial

Table of Contents

Appendix 1a. Cumulative incidence of locally acquired malaria 2
Appendix 1b. Prevalence of quantitative PCR-detected infection 3
Appendix 2. Comparison groups 4

For peer review only

Appendix 1a. Cumulative incidence of locally acquired malaria

	No. of EAs	Incidence per 1000 person-years (95% CI)	p value	Incidence rate ratio (95% CI)	p value	Adjusted incidence rate ratio (95% CI)	p value
**Human reservoir**							
RACD	27	38.3 (23.0–53.6)	0.26	1 (reference)	0.51	1 (reference)	0.009
rfMDA	28	30.8 (12.8–48.7)		0.82 (0.26–1.37)		0.52 (0.16–0.88)	
**Mosquito reservoir**							
No RAVC	27	38.9 (20.7–57.1)	0.23	1 (reference)	0.41	1 (reference)	0.002
RAVC	28	30.2 (15.0–45.5)		0.78 (0.26–1.30)		0.48 (0.16–0.80)	
**Mosquito and human reservoir**							
RACD only	13	41.4 (21.5–61.2)	0.11	1 (reference)	0.32	1 (reference)	0.006
rfMDA+RAVC	14	25.0 (5.2–44.7)		0.62 (0.24–1.59)		0.26 (0.10–0.68)	

RACD: reactive case detection, rfMDA: reactive focal mass drug administration, RAVC: reactive vector control

For peer review only

Appendix 1b. Prevalence of quantitative PCR-detected infection

	N	Prevalence (95% CI)	p value	Prevalence ratio (95% CI)	p value	Adjusted prevalence ratio (95% CI)	p value
**Human reservoir**							
RACD	2150	3.78% (2.85-5.00)	0.46	1 (reference)	0.92	1 (reference)	0.009
rfMDA	1932	3.16% (2.14-4.65)		1.05 (0.03-2.07)		0.59 (0.21-0.98)	
**Mosquito reservoir**							
No RAVC	2030	4.07% (2.92-5.64)	0.15	1 (reference)	0.13	1 (reference)	0.002
RAVC	2052	2.92% (2.13-3.99)		0.61 (0.10-1.12)		0.36 (0.13-0.59)	
**Human and mosquito reservoir**							
RACD only	1016	3.70% (2.39-5.69)	0.04	1 (reference)	0.17	1 (reference)	0.004
rfMDA+RAVC	918	1.75% (0.99-3.09)		0.52 (0.20-1.32)		0.16 (0.05-0.55)	

RACD: reactive case detection, rfMDA: reactive focal mass drug administration, RAVC: reactive vector control

For peer review only

Appendix 2. Comparison groups

**rfMDA *versus* RACD**			Human reservoir	
			**RACD (28 clusters)**	**rfMDA (28 clusters)**
	Mosquito reservoir	**No RAVC**	RACD only	rfMDA only
		**RAVC**	RACD + RAVC	rfMDA + RAVC

**RAVC *versus* No RAVC**			Human reservoir	
			**RACD**	**rfMDA**
	Mosquito reservoir	**No RAVC (28 clusters)**	RACD only	rfMDA only
		**RAVC (28 clusters)**	RACD + RAVC	rfMDA + RAVC

**rfMDA +RAVC *versus* RACD only**			Human reservoir	
			**RACD**	**rfMDA**
	Mosquito reservoir	**No RAVC**	RACD only **(14 clusters)**	rfMDA only
		**RAVC**	RACD + RAVC	rfMDA + RAVC **(14 clusters)**

RACD: Reactive case detection, RAVC: Reactive vector control
rfMDA: Reactive focal Mass Drug administration

For peer review only

BMJ Open

Cost and cost-effectiveness of reactive case detection (RACD), reactive focal mass drug administration (rfMDA), and reactive focal vector control (RAVC) to reduce malaria in the low endemic setting of Namibia: an analysis alongside a 2x2 factorial design cluster randomised controlled trial

Journal:	*BMJ Open*
Manuscript ID	bmjopen-2021-049050.R1
Article Type:	Original research
Date Submitted by the Author:	20-Apr-2022

Complete List of Authors:	Ntuku, Henry; University of California San Francisco, Global Health Group, Malaria Elimination Initiative<br>Smith-Gueye, Cara; University of California San Francisco, Malaria Elimination Initiative<br>Scott, Valerie; University of California San Francisco, Malaria Elimination Initiative<br>Njau, Joseph; JoDon Consulting Group, LLC<br>Whittemore, Brooke; The University of Texas Southwestern Medical Center, Department of Pediatrics<br>Zelman, Brittany; University of California San Francisco, Malaria Elimination Initiative<br>Tambo, Munyaradzi; University of Namibia, Multidisciplinary Research Centre<br>Prach, Lisa; University of California San Francisco, Malaria Elimination Initiative<br>Wu, Lindsey; London School of Hygiene & Tropical Medicine, Department of Infection Biology<br>Schrubbe, Leah; University of California San Francisco, Malaria Elimination Initiative<br>Kang Dufour, Mi-Suk; University of California San Francisco, Division of Prevention Science<br>Mwilima, Agnes; Zambezi Ministry of Health and Social Services<br>Uusiku, Petrina; Namibia Ministry of Health and Social Services, National Vector-Borne Diseases Control Program<br>Sturrock, Hugh; University of California San Francisco, Malaria Elimination Initiative<br>Bennett, Adam; University of California San Francisco, Malaria Elimination Initiative, Global Health Group<br>Smith, Jennifer; University of California San Francisco, Malaria Elimination Initiative<br>Kleinschmidt, Immo; University of the Witwatersrand, School of Pathology, Faculty of Health Sciences; London School of Hygiene & Tropical Medicine, Department of Infectious Disease Epidemiology<br>Mumbengegwi, Davis; University of Namibia, Multidisciplinary Research Centre

	Gosling, Roly; University of California San Francisco, Global Health Group<br>Hsiang, Michelle; University of California San Francisco, Malaria Elimination Initiative; The University of Texas Southwestern Medical Center, Department of Pediatrics
**Primary Subject Heading**:	Health economics
Secondary Subject Heading:	Health policy, Public health, Epidemiology, Global health, Infectious diseases
Keywords:	EPIDEMIOLOGY, HEALTH ECONOMICS, Health policy < HEALTH SERVICES ADMINISTRATION & MANAGEMENT, Public health < INFECTIOUS DISEASES

SCHOLARONE™
Manuscripts

I, the Submitting Author has the right to grant and does grant on behalf of all authors of the Work (as defined in the below author licence), an exclusive licence and/or a non-exclusive licence for contributions from authors who are: i) UK Crown employees; ii) where BMJ has agreed a CC-BY licence shall apply, and/or iii) in accordance with the terms applicable for US Federal Government officers or employees acting as part of their official duties; on a worldwide, perpetual, irrevocable, royalty-free basis to BMJ Publishing Group Ltd ("BMJ") its licensees and where the relevant Journal is co-owned by BMJ to the co-owners of the Journal, to publish the Work in this journal and any other BMJ products and to exploit all rights, as set out in our licence.

The Submitting Author accepts and understands that any supply made under these terms is made by BMJ to the Submitting Author unless you are acting as an employee on behalf of your employer or a postgraduate student of an affiliated institution which is paying any applicable article publishing charge ("APC") for Open Access articles. Where the Submitting Author wishes to make the Work available on an Open Access basis (and intends to pay the relevant APC), the terms of reuse of such Open Access shall be governed by a Creative Commons licence – details of these licences and which Creative Commons licence will apply to this Work are set out in our licence referred to above.

Other than as permitted in any relevant BMJ Author's Self Archiving Policies, I confirm this Work has not been accepted for publication elsewhere, is not being considered for publication elsewhere and does not duplicate material already published. I confirm all authors consent to publication of this Work and authorise the granting of this licence.

For peer review only

Cost and cost-effectiveness of reactive case detection (RACD), reactive focal mass drug administration (rfMDA), and reactive focal vector control (RAVC) to reduce malaria in the low endemic setting of Namibia: an analysis alongside a 2x2 factorial design cluster randomised controlled trial

Henry Ntuku, PhD[1], Cara Smith Gueye, PhD[1], Valerie K. Scott, MSPH[1], Joseph Njau, PhD[2], Brooke Whittemore, MPH[3], Brittany Zelman, MPH[1], Munyaradzi Tambo, PhD[4], Lisa M. Prach, PhD[1], Lindsey Wu, PhD[5], Leah Schrubbe, MSc[1], Mi-Suk Kang Dufour, PhD[6], Agnes Mwilima, BA[7], Petrina Uusiku, MD[8], Hugh Sturrock, PhD[1], Adam Bennett, PhD[1], Jennifer L. Smith, PhD[1], Prof Immo Kleinschmidt, PhD[9,10,12], Prof Davis Mumbengegwi, PhD[4], Prof Roly Gosling, PhD[1], Michelle S. Hsiang, MD[1,3, 11]

[1]Malaria Elimination Initiative, Global Health Group, University of California San Francisco (UCSF), USA; [2]JoDon Consulting Group, LLC; Atlanta, GA , USA; [3]Department of Pediatrics, University of Texas Southwestern Medical Center, Dallas, TX, USA; [4]Multidisciplinary Research Centre, University of Namibia, Windhoek, Namibia; [5]Department of Infection Biology, London School of Hygiene and Tropical Medicine, [6]Division of Prevention Science, UCSF, USA; [7]Zambezi Ministry of Health and Social Services, Katima, Namibia; [8]National Vector-Borne Diseases Control Program, Ministry of Health and Social Services, Windhoek, Namibia; [9]School of Pathology, Faculty of Health Sciences, University of the Witwatersrand, Johannesburg, South Africa; [10]Department of Infectious Disease Epidemiology, London School of Hygiene and Tropical Medicine, London, UK; [11]Department of Pediatrics, UCSF, USA; [12]Southern African Development Community Malaria Elimination Eight Secretariat, Windhoek, Namibia.

*Correspondence to: Henry Ntuku MD PhD, Malaria Elimination Initiative, Global Health Group, University of California San Francisco (UCSF), 550 16th Street, 3rd Floor San Francisco, CA 94158, USA.

hntuku@path.org

Word count: 4282

For peer review only

ABSTRACT

Objectives

To estimate the cost and cost-effectiveness of reactive case detection (RACD), reactive focal mass drug administration (rfMDA), and reactive focal vector control (RAVC) to reduce malaria in a low endemic setting.

Setting

The study was part of a 2x2 factorial design cluster randomised controlled trial within the catchment area of 11 primary health facilities in Zambezi, Namibia.

Participants

Cost and outcome data were collected from the trial, which included 8948 community members that received interventions due to their residence within 500 meters of malaria index cases.

Outcome measures

The primary outcome was incremental cost effectiveness ratio (ICER) per in incident case averted. ICER per prevalent case and per disability adjusted life years (DALY) averted were secondary outcomes, as were per unit interventions costs (USD) and personnel time. Outcomes were compared as: 1) rfMDA *versus* RACD, 2) RAVC *versus* No RAVC and 3) rfMDA+RAVC *versus* RACD only.

Results

rfMDA cost 1.1x more than RACD, and RAVC cost 1.7x more than no RAVC. Relative to RACD only, the cost of rfMDA+RAVC was double ($3,082 *versus* $1,553 per event). The ICERs for rfMDA *versus* RACD, RAVC *versus* No RAVC, and rfMDA+RAVC *versus* RACD only were $114, $1,472, and $842, per incident case averted, respectively. Using prevalent infections and DALYs as outcomes, trends were similar. The median personnel time to implement rfMDA was 20% lower than for RACD (30 *versus* 38 minutes per person). The median personnel time for RAVC was 34 minutes per structure sprayed.

Conclusion

Implemented alone or in combination, rfMDA and RAVC were cost-effective in reducing malaria incidence and prevalence despite higher implementation costs in the intervention compared to control arms. Compared to RACD, rfMDA was time-saving. Cost and time requirements for the combined intervention could be decreased by implementing rfMDA and RAVC simultaneously by a single team.

Keywords: malaria, malaria elimination, Plasmodium falciparum, cost-effectiveness, economic, reactive, active case detection, mass drug administration, focal, indoor residual spraying, vector control, Anopheles

For peer review only - http://bmjopen.bmj.com/site/about/guidelines.xhtml

Strengths and limitations of this study:

- This is the first study to evaluate the costs and cost effectiveness of two innovative malaria transmission reducing strategies, reactive focal mass drug administration and reactive focal vector control, used alone and in combination.

- Estimates are derived from prospective data collection of costs and robust measures of effectiveness provided by a rigorous community randomised controlled trial.

- In a secondary analysis, the study interventions were also found to be cost-effectiveness to avert disability-adjusted life-years (DALYs).

- The study design did not allow comparison of each intervention alone, since half of the clusters in each study arm compared received another intervention.

INTRODUCTION

In recent years, progress in malaria control and elimination, using standard approaches, has faltered [1], leading to plateuing case numbers abd seasonal outbreaks [2]. New malaria elimination approaches are needed, yet there are limited data on their costs and cost-effectiveness. In a context of declining donor funding, insufficient human resources and competing health priorities, economic evidence regarding malaria elimination interventions is needed to guide decision-making.

In areas with declining transmission intensity, a large proportion of infections are asymptomatic and tend to cluster geographically and temporally [3]–[5]. Since malaria vectors are still present, these infections may seed further transmission in their immediate neighborhood, leading to focal outbreaks or clusters of malaria cases [6]. To prevent wider epidemics, community-based targeted interventions in response to passively identified malaria cases are recommended and implemented by programs aiming to interrupt transmission [7], [8]. One widely implemented intervention is reactive case detection (RACD), whereby individuals residing near passively detected index cases within a pre-defined radius are screened with a malaria rapid diagnostic test (RDT) and treated if results are positive. However, RACD may have limited impact because RDTs fail to identify low density parasite infections, which

For peer review only - http://bmjopen.bmj.com/site/about/guidelines.xhtml

predominate in low transmission settings [4], [9]–[12]. To Circumvent this challenge of missed low-density infections, reactive focal mass drug administration (rfMDA) is an alternative approach in which the same household members and neighbors of index cases are presumptively treated with an antimalarial drug irrespective of infection status [13]. Reactive focal vector control (RAVC) is another promising elimination strategy, where focal indoor residual spraying (IRS) is targeted to households near index cases identified through passive case detection [13]. RAVC ensures sufficient IRS coverage in the areas at highest risk of malaria transmission. These households may have been insufficiently covered during a national IRS campaign, typically held months before the malaria season starts. The combination of rfMDA and RAVC has been shown to be more effective than the interventions administered alone [13].

In addition to an intervention's effectiveness in reducing transmission, costs and cost-effectiveness are key considerations in the decision-making process for National Malaria Control Programs and countries embarking on malaria elimination. Given that malaria control budgets are limited, interventions or their combinations must be chosen based on expected cost and impact. While a few studies have examined costs associated with RACD [14], [15], there is very little information available on economic evaluations of rfMDA. The limited data available report on the costs of focal mass drug administration following community-wide screening [16] or mass drug administration (MDA), which is typically administered as a mass campaign [17]. Compared to standard IRS, RAVC has been shown to be cost-effective in a very low transmission setting [18]. To our knowledge there are no published economic data on RAVC, nor rfMDA+RAVC, in a low transmission setting.

This paper reports the findings of an economic evaluation of three reactive strategies – RACD, rfMDA and RAVC – carried out as part of a cluster randomised controlled trial (CRCT) conducted in Zambezi Region, Namibia in 2017 [13]. The goal of the trial was to investigate the effectiveness and feasibility of novel interventions that target the parasite reservoir in humans (rfMDA) and the vector

For peer review only - http://bmjopen.bmj.com/site/about/guidelines.xhtml

(RAVC) and are applied in a reactive and focal fashion (e.g. in response to confirmed, passively identified malaria cases). Costs of these interventions were compared singly and in combination to their respective current standard of care (e.g. rfMDA *vs* RACD, RAVC *vs* no RAVC, and rfMDA+RAVC *vs* RACD+no RAVC). Personnel time required for the interventions were also compared.

METHODS

Study site and description of the trial

The study was conducted from January to December 2017 in Zambezi region, northern Namibia. From 2010 to 2015, annual parasite incidence was low (<15/1000 population), likely due to an increase in malaria funding and scaling up of effective interventions including improved case management and wide-scale implementation of preseason IRS campaigns with dichlorodiphenyltrichloroethane (DDT) or deltamethrin [19]. However, there was a resurgence in the year prior to the trial (2016) and incidence rose to 33/1,000 population. Transmission is almost entirely due to *Plasmodium falciparum*. *Anopheles arabiensis* is the dominant vector, with the high transmission season occurring from January to June.

As reported elsewhere, the parent study was a CRCT with 2x2 factorial design [13][20]. In brief, 56 census enumeration areas (EAs) within the catchment area of 11 health facilities were randomised to receive rfMDA or RACD, and RAVC or no RAVC, resulting in four study arms: 1) RACD only, 2) RACD + RAVC, 3) rfMDA only, 4) rfMDA+RAVC (Table 1). The primary outcome was cumulative incidence of local malaria cases and the secondary outcome was infection prevalence as assessed in an endline survey. The total study population was 18,803 individuals. Study interventions were triggered by an index case diagnosed at the study health facilities by either RDT or microscopy and reported through an electronic rapid reporting system. Within approximately two weeks of reporting, a study team consisting of a field investigator, nurse, and driver/data collector visited the index case household and neighboring households within a 500-meter radius with a target of enrolling at least 25 individuals for RACD and

For peer review only - http://bmjopen.bmj.com/site/about/guidelines.xhtml

rfMDA. For RAVC, a separate team consisting of a spray team leader/driver and a spray assistant visited the same location and sprayed the index case household and seven closest households. For each intervention, one follow-up visit was made if the enrolment target was not reached at the initial visit.

RACD consisted of testing for malaria using an RDT (CareStart Malaria HRP2/pLDH Pf/PAN, Access Bio, Somerset, New Jersey, USA) and treating those with positive results with artemether-lumefantrine (AL) (Coartem®, Novartis, Switzerland) and single low dose primaquine (Primaquine, Remedica, Cyprus) per national policy. Dried blood spots (DBS) were collected for molecular analysis. For rfMDA, eligible individuals were offered a presumptive, weight-appropriate, 3-day treatment course of AL only (Coartem®, Novartis, Switzerland). RAVC consisted of spraying ceilings and walls of sleeping structures with pirimiphos-methyl (Actellic 300 CS, Syngenta AG, Basel, Switzerland) per standard procedures [21]. To assess medication adherence, the team performed a follow-up pill count 7–10 days after the intervention for all RACD participants who were prescribed AL and among a sample of rfMDA participants.

The results of the trial showed that rfMDA and RAVC implemented alone and in combination reduced malaria incidence and parasite prevalence. Specifically, rfMDA and RAVC each reduced malaria incidence by nearly 50% when compared with their respective controls RACD and no RAVC, and their combination reduced malaria incidence by 75% compared to RACD only. Similarly, rfMDA and RAVC reduced prevalence by 41% and 64%, compared to their respective controls RACD and no RAVC and their combination reduced prevalence by 84% compared to RACD only [13].

Collection of costing data

The costing analysis was undertaken from the provider or program perspective. An ingredients-based or micro-costing approach was used for line-item cost estimation. Inputs were identified, quantified, valued and classified into activity category [22]. Cost data were collected retrospectively from the

For peer review only - http://bmjopen.bmj.com/site/about/guidelines.xhtml

financial expenditure records. No adjustments for inflation were made. Costs of items were valued according to their market values in the year when they were purchased, in either Namibia dollars (NAD) or U.S. dollars (USD). Costs in NAD were converted to USD using the official 2017 average exchange rate of 1 USD per 14.28 NAD [23]. Data were entered into a costing tool in Microsoft Excel 2010.

Costs were classified as either capital or recurrent, showing the difference between investment once-off costs versus those that represent the running costs of ongoing program implementation. Capital costs included vehicles, computers, tablets, printers, photocopiers, and office furniture. For capital items such as vehicles, office furniture, and computers estimated to have life years beyond the study period, a discount rate of 3% and depreciation were applied before including one year value of the equipment to the costs of the study (estimated project evaluation duration) [24]. Recurrent costs were grouped into one of four categories: consumables, services, personnel, or training and meetings. Consumables included field supplies and commodities related to the study intervention (e.g. RDTs, drugs and insecticides). Services included fuel for vehicles, airtime for staff, data bundles for tablets, maintenance of equipment, and office rental. Personnel costs included salaries and fringe benefits at a partner pay scale. Based on the time and motion observation (see below), a percentage of the personnel time spent on each intervention was applied to the full annual costs (including salary, benefits, and any types of allowances or per diems). Costs were assigned to either RACD, rfMDA, or RAVC. For items shared by different intervention types, a percentage of use was allocated based on trial implementation data. The costing assessment was limited to the basic intervention level to resemble programmatic costs as close as possible. Costs related to research (i.e. international collaboration, the endline survey or molecular analyses) were excluded.

Personnel time

To assess the average time spent to complete the different interventions, a time and motion approach was used [25]. A field coordinator observed field personnel participating in the event, and recorded time

For peer review only - http://bmjopen.bmj.com/site/about/guidelines.xhtml

spent by each staff member performing specific tasks. The sum of time spent by each personnel on each task was used to compute the total average time to complete the event. Data on personnel time were collected as part of supervisory visits for a sample of 10 events for each strategy (rfMDA, RACD, and RAVC). Observed activities included: preparation time in the office, driving time, and event start and end times at the village. Data regarding the number of people treated (rfMDA) or tested and treated if positive (RACD) or the total number of structures sprayed (RAVC) were obtained from the trial database.

Collection of outcome data

Data on population at risk by study arms (defined as the total population in the study arm during the study period), number of events conducted, number of households sprayed and the total population residing in the sprayed households, number of people tested or presumptively treated, number of RDT-detected infections during RACD, and malaria case incidence and infection prevalence data were retrieved from the trial database [13] (**Table 1 and Appendix 1**).

Data Analysis

The following three comparison groups were used to compare intervention to control arms and evaluate the cost and cost-effectiveness of interventions targeting the parasite reservoir: in humans, in mosquitoes, and in both humans and mosquitoes, respectively (3) (**Appendix 2**): (1) rfMDA *versus* RACD, (2) RAVC *versus* no RAVC and (3) rfMDA+RAVC *versus* RACD only. Costing data reflect the way that interventions were implemented in the 2x2 factorial design. Specifically, for the rfMDA *versus* RACD comparison, expenses for the rfMDA group included those from the rfMDA only and rfMDA+RAVC arms, and expenses for the RACD group included those from the RACD only and RACD+RAVC arms. The same approach was used for the RAVC *versus* no RAVC comparison, in which all expenses occurring in the RAVC arm (RACD+RAVC and rfMDA+RAVC) were compared to costs occurring in the no RAVC arm (RACD only and rfMDA only). With this approach, the no RAVC arm had non-zero costs and could be compared

For peer review only - http://bmjopen.bmj.com/site/about/guidelines.xhtml

For peer review only

to RAVC. For the rfMDA+RAVC *versus* RACD only, the total costs of the combined interventions were compared to the total costs of RACD only.

To compare the costs, the following cost outcomes indicators were calculated: (1) total cost of the intervention for the duration of the one-year trial; (2) cost per intervention event, calculated as the total cost of the strategy divided by the total number of responses conducted; (3) cost per individual, calculated as the total cost of the strategy divided by either the total number of persons tested (for RACD), the total number of persons treated (for rfMDA), or the total number of persons residing in households sprayed (for RAVC); (4) cost per population at risk (PAR), calculated as the total cost of the intervention divided by the total population in the study arm in which the specific intervention was implemented.

For the cost of rfMDA and RAVC individually, we report costs relative to the control group because half of the clusters in these groups received a different intervention than the one being evaluated (i.e half of the EAs in the rfMDA group received RAVC and in the RAVC group half of the EAs received RACD and the other half received rfMDA), resulting in challenges to assess the cost and cost-effectiveness of actual individual interventions. For the cost of rfMDA+RAVC combined, we report exact figures because the analyses are restricted to EAs receiving these specific interventions (**Appendix 2**).

For cost effectiveness, the incremental cost effectiveness ratio (ICER) was calculated as the difference in costs between intervention and control divided by the difference in their effect: *ICER= [(Cost of intervention) - (Cost of control)]/ [Effect of intervention – Effect of control].* For the effect, we used three different impact measures; incidence rates (ICER per incident case averted), infection prevalence (ICER per prevalent infection averted), and disability adjusted life years DALYs (ICER per DALY averted). Models used to estimate adjusted incidence rate ratios and adjusted prevalence ratios with 95% confidence intervals (CIs), as reported in [13], were used to predict incidence or infection prevalence. Using the population of the study area, the number of incident cases and prevalent

For peer review only - http://bmjopen.bmj.com/site/about/guidelines.xhtml

infections was then estimated and used to calculate ICERs. DALYs averted were estimated using a mean loss of 1.18 DALYs per malaria case [26] with disability weights and life expectancy of 63.5 years for females and 58.9 years for males from the World Health Organization (WHO) life tables [27], and an average duration of 7 days for a malaria episode without age weighting and discounting [28].

To define the interventions as cost effective, we used Namibia's 2017 gross domestic product (GDP) per capita[29] and the WHO economic evaluation guidelines on cost effectiveness thresholds for low- and middle-income countries. The guidelines consider any program costs that are less than the national per capita GDP per DALY averted to be highly cost effective. Program costs that are less than three times the national per capita GDP per DALY averted are considered cost effective [30].

To enable comparisons between study arms, personnel time is presented as time to complete an intervention event (from arrival at index case household to departure from village of index case) excluding preparation time and driving time.

Sensitivity Analysis

A three-point estimation (PERT) sensitivity analysis was performed to validate the cost-effectiveness outcome results. This analysis focused on varying the effectiveness of the interventions by varying malaria incidence using the lower and upper limit of the confidence interval of predicted incidence in interventions arms while keeping median estimates unchanged for control arms.

The discount rate, personnel costs, and consumables costs were also varied, and results obtained were compared to median estimates hereby referred to as best estimates. The discount rate was varied between 0 and 5%. For personnel, the governmental salary pay scale (appendix 3) for all staff (nurses, spray operators, drivers, field supervisors, etc.) was used. Also, as malaria testing and treatment and IRS can be conducted by trained community health workers (CHWs), their compensation (standard rate of 260 NAD ($18.20 USD) as daily labor costs) was also considered. Finally, for the commodity price

For peer review only - http://bmjopen.bmj.com/site/about/guidelines.xhtml

analysis, which was only used for RAVC, the government's subsidized price of $15 per bottle of pirimiphos-methyl (Actellic 300 CS) was considered.

Patient and Public involvement

Neither patients nor members of the public were involved in the design, conduct, reporting, or dissemination plans of the study.

RESULTS

During trial implementation, a total of 342 intervention events were conducted and 8,948 individuals received RACD or rfMDA, and RAVC or no RAVC (mean 26 participants/event). The total numbers of events conducted and individuals receiving each intervention by assigned group, and by comparison arm are shown in **Table 1.**

The total cost of conducting rfMDA was 1.1x higher than RACD, while RAVC cost 1.7x more than no RAVC. The costs of rfMDA+RAVC were 1.8x higher than RACD only ($234,223 *versus* $127,312) (**Table 2**). Personnel costs were the main cost driver for all three interventions, accounting for 66% of the RACD costs, 62% of rfMDA costs and 64% of RAVC costs (**Figure 1**). As shown in **Table 2** and **Figure 2,** for all comparison groups, unit costs were higher in intervention groups compared to controls. The cost per population at risk was 1.2x higher for rfMDA compared to RACD ($41.40 *versus* $35.80), 1.7x higher for RAVC compared to no RAVC ($48.80 *versus* $28.00) and two times higher for rfMDA+RAVC than RACD only ($54.40 *versus* $26.90). In the RACD arms, there was a total of 114 infections identified, which translated to $3,112 per infection identified.

Using incidence and prevalence outcome data from the trial (**Appendices 1a and 1b**), the number of malaria incident cases and prevalent infections averted among study arms population were estimated, and ICERs were calculated for each comparison group to evaluate cost effectiveness. The

For peer review only - http://bmjopen.bmj.com/site/about/guidelines.xhtml

base case scenario results for impact and cost effectiveness are shown in **Table 3**. Compared to RACD only, rfMDA+RAVC had an ICER of $842 per incident case averted and $1,812 per prevalent infection averted. The implementation of rfMDA resulted in an ICER of $114 per incident case averted and $162 per prevalent infection averted compared to RACD. RAVC was associated with an ICER of $1,472 per incident case averted and $2,670 per prevalent infection averted compared to no RAVC (**Figure 3**).

Using mean DALYs loss estimates [28] and the number of incident cases averted, the implementation of rfMDA and RAVC translated into an ICER of $97 per DALY averted for rfMDA compared to RACD, $1,248 per DALY averted for RAVC compared to no RAVC, and $714 per DALY averted for rfMDA+RAVC compared to RACD only (**Figure 3**). Given that Namibia's 2017 GDP per capita was $6,193 [29], both rfMDA and RAVC as implemented in Zambezi region, singly or combined would be considered highly cost-effective health interventions.

Sensitivity analysis

The parameters included in the sensitivity analysis and the change in costs and ICERs are detailed in **Table 4** and **Figure 4**. For all three comparison groups, the ICER per incident case averted were sensitive to variations in predicted malaria incidence, and in personnel salary scale. Costs per PAR were sensitive to variations in personnel salary scale. Within the range of the variation used, discount rate and commodity cost (for Actellic 300 CS) did not have a significant impact on cost per PAR nor ICERs.

Personnel time

Data regarding personnel time by intervention are shown in **Table 5**. The time spent by teams to enroll a participant was longer for RACD compared to rfMDA with a median personnel-minutes per participant enrolled of 38 minutes (IQR: 35–44) versus 30 minutes (IQR: 25–38). The median personnel-minutes per structure sprayed for RAVC was 34 minutes (IQR: 29–38). With an average of 4 people per household,

For peer review only - http://bmjopen.bmj.com/site/about/guidelines.xhtml

the median personnel-minutes per individual protected with RAVC was 9 minutes. The median preparation time was 33 minutes for rfMDA and 32 minutes for RACD and RAVC. For all interventions, the teams spent on average 2 hours per event travelling to and from the index case household.

DISCUSSION

Results of this study show that compared to RACD, reactive focal interventions (rfMDA, RAVC, and rfMDA+RAVC combined) used in a low transmission Sub-Saharan African setting, are cost effective, when considering outcomes of malaria incidence or prevalence, or DALYs averted. In addition, rfMDA was time saving compared to RACD. These results, taken together with the main trial findings on effectiveness of these interventions, suggest that in such settings, a change in policy and practice from RACD to rfMDA and/or RAVC should be considered [11].

It is established that the investment needed to achieve malaria elimination will be higher per case than that of standard malaria control [31], [32]. For example, the costs per person reported in this study for RAVC ($48.80) are significantly higher than the ones reported by White *et al* in a systematic review on costs and cost-effectiveness of malaria control interventions ($2.20 for mosquito nets and $6.70 for IRS)[33]. However, in the elimination context, a comparison to the current standard of care, which often includes RACD, is more useful. Further, it is generally accepted that the long-term benefits of elimination outweigh the costs, making the pursuit worthwhile [31], [32].

Although rfMDA costs are marginally higher compared to RACD, the difference is mostly a reflection of economies of scale. Due to the way in which we have calculated costs in relation to events, we show a conservative assessment of costs and cost-effectiveness. For example, because RACD was less effective, more RACD events were carried out and more people tested, lowering the cost per person. In the only other reported study of costs for RACD, conducted by Zelman et al. in Indonesia [34],

For peer review only - http://bmjopen.bmj.com/site/about/guidelines.xhtml

the cost per individual screened, without major start-up capital costs, was lower ($28 compared to $39 in this study) but that study had more individuals enrolled per RACD event (42 individuals compared to 25 individuals per event in this study). Also, in this study, the total costs of the combined intervention and RACD only should not be compared to those of the individual interventions because the combination intervention was only conducted in half the study clusters.

In the sensitivity analyses, both rfMDA and RAVC implemented alone or in combination remained cost effective with varying effectiveness. The sensitivity analysis also provided an indication on how the costs of interventions may be reduced in the future. Costs could be reduced by more than 10% if the interventions are to be implemented by the ministry of health using existing resources (e.g. personnel, vehicles, fuel, etc.) (**Table 4**). Costs could be further reduced through better integration of staff with other disease control programs. Consistent with other studies of malaria community level interventions [35], [36], personnel costs were the main cost driver. Conducting reactive interventions is a challenging and resource intensive task, requiring dedicated teams working throughout the malaria transmission season to achieve high coverage of interventions. A CHW program could be considered as a potentially cost-effective approach for the delivery of health services to communities [37]. CHWs are used in similar settings to deliver reactive interventions[16], [38], [39], and Namibia currently has a pool of trained and accredited CHWs to test and treat malaria who could be used in the implementation of reactive interventions. A community-based, self-administered treatment approach using village health workers is currently being tested in a randomised controlled trial in the Gambia as a cost effective way to deliver rfMDA [40]. Finally, considerable cost savings can be made by combining rfMDA or RACD teams and activities with RAVC, which we did not do in the study due to protocol design.

Despite the time requirements associated with rfMDA, such as reviewing potential contraindications and adverse effects, and ensuring treatment compliance, the time required to implement rfMDA was less, due to the time it takes to conduct an RDT, wait for the results, share the

For peer review only - http://bmjopen.bmj.com/site/about/guidelines.xhtml

result with the participant, and then provide treatment if indicated. Not surprisingly, the median personnel-time per participant was shorter in the rfMDA arms compared to RACD. The shorter time per participant in rfMDA is expected to result in a higher number of individuals covered by rfMDA in a program implementation setting, potentially leading to a lower cost per person due to economies of scale.

This study was limited in several ways. The intervention was not designed to minimize costs and maximize cost effectiveness. For example, in rfMDA arms, more than the targeted number of 25 individuals could have been enrolled per event with the same staff working time and could have resulted in reduced costs per person enrolled. The study design did not allow direct comparison between actual single interventions, since half of the EAs in the RACD and the rfMDA comparison groups received RAVC, and for RAVC, half of the EAs received RACD and half received rfMDA. Although all efforts were made to exclude costs related to research activities, it was not possible to identify the costs related to some research activities such as time needed to obtain informed consent, conduct interviews, and collect research related blood samples, and therefore, these costs could not be excluded from the analysis. Program implementation costs of these interventions are therefore likely to be lower. Moreover, the inclusion of the wider social and economic benefits of malaria prevention (lost wages, direct healthcare costs, transport costs to seek care), though beyond the scope of this work, would likely result in higher estimates of cost-effectiveness. Finally, we limited our assessment to the test interventions used in the trial, and used RACD and/or no RAVC as a comparison. It is possible that comparisons with other approaches, including non-targeted interventions, and/or use of a different control (e.g. no RACD), would also be cost-effective[41].

In conclusion, in the low transmission setting of Namibia, rfMDA and RAVC implemented as singly or in combination are highly cost-effective interventions that can substantially reduce malaria transmission. Compared to RACD, rfMDA was time-saving, and for the combination intervention, the

For peer review only - http://bmjopen.bmj.com/site/about/guidelines.xhtml

additional cost and time requirements for RAVC could be minimized by having a single team implement both rfMDA and RAVC at the same time. Given the frequent occurrence of outbreaks in low transmission areas of southern Africa, applying the rfMDA +/- RAVC approach may be effective at bringing outbreaks rapidly under control. These results offer a good indication of the best value for money, though additional research is needed to provide information on other factors to guide decision making such as affordability and budget impact. Beyond ICERs, the net benefits framework provides additional guidance to decision-makers for the choice of interventions using the probability of the intervention to achieve elimination of transmission [42]. Findings are likely to be generalizable to other low transmission settings where cases of malaria are highly clustered around index cases.

For peer review only - http://bmjopen.bmj.com/site/about/guidelines.xhtml

Table 1. Main trial design and costing outcome measures

		Human reservoir	
		**RACD**<br>(reactive case detection)<br>- 28 clusters<br>- Pop at risk: 9898<br>- No of events: 178<br>- No of people: 4701<br>- No of RDT identified infections: 114	**rfMDA**<br>(reactive focal mass drug administration)<br>- 28 clusters<br>- Pop at risk: 8905<br>- No of events: 164<br>- No of people: 4247
Mosquito reservoir	**No RAVC** (no reactive focal vector control)<br>- 28 clusters<br>- Pop at risk: 9339<br>- No of events: 170<br>- No of people: 4369	**RACD only arm**<br>- 14 clusters<br>- Pop at risk: 4742<br>- No of events: 82<br>- No of people: 2188<br>- No of RDT identified infections: 52	**rfMDA only arm**<br>- 14 clusters<br>- Pop at risk: 4597<br>- No of events: 88<br>- No of people: 2181
	**RAVC** (reactive focal vector control)<br>- 28 clusters<br>- Pop at risk: 9464<br>- No of events: 172<br>- No of people: 4032	**RACD + RAVC arm**<br>- 14 clusters<br>- Pop at risk: 5156<br>- No of events: 96<br>- No of people: 2513<br>- No of RDT identified infections: 62	**rfMDA + RAVC arm**<br>- 14 clusters<br>- Pop at risk: 4308<br>- No of events: 76<br>- No of people: 2066

For peer review only - http://bmjopen.bmj.com/site/about/guidelines.xhtml

Table 2. Total and unit costs per comparison group (Cost in 2017 USD)

	Average costs (Start-up and recurrent)					Recurrent costs only				
Comparison groups	Total costs	Cost per event	Cost per person	Cost per PAR#	Cost per infection identified	Total costs	Cost per event	Cost per person	Cost per PAR	Cost per infection identified
Human reservoir										
RACD*	$354,750	$1,993	$75.5	$35.8	$3,112	$309,368	$1,738	$65.8	$31.3	$2,714
rfMDA†	$368,321	$2,246	$86.7	$41.4	-	$322,940	$1,969	$76.1	$36.3	-
Mosquito reservoir										
No RAVC‡	$261,409	$1,537	$59.8	$28.0	-	$227,837	$1,340	$52.2	$24.4	-
RAVC	$461,661	$2,684	$114.5	$48.8	-	$404,470	$2,352	$100.3	$42.7	-
Human and mosquito reservoir										
RACD only	$127,312	$1,553	$58.2	$26.9	$2,448	$110,526	$1,348	$50.5	$23.3	$2,126
rfMDA+RAVC	$234,223	$3,082	$113.4	$54.4	-	$205,628	$2,706	$99.5	$47.7	-

#PAR: population at risk, *RACD: Reactive case detection, † rfMDA: Reactive focal Mass Drug administration, ‡RAVC: Reactive focal vector control

For peer review only - http://bmjopen.bmj.com/site/about/guidelines.xhtml

Table 3. Impact indicators and incremental cost effectiveness ratio (ICER) per comparison group (cost in 2017 USD)

Comparison groups	Predicted incidence per 1000 person-years (95% CI)	No. of Incident cases	No. of Incident cases averted	DALYs averted	Predicted infection prevalence (95% CI)	No. of Prevalent infections	No. of Prevalent infections averted	ICER per incident case averted	ICER per prevalent infection averted	ICER per DALY averted
Human reservoir										
RACD	42.1 (30.0–54.2)	396	Ref	Ref	3.3% (2.7–4.0)	310	Ref	Ref	Ref	Ref
rfMDA	29.5 (19.0–40.0)	277	119	140	2.4% (1.6–3.2)	226	84	$114	$162	$97
Mosquito reservoir										
No RAVC	43.1 (28.7–57.6)	405	Ref	Ref	3.5% (2.7–4.3)	329	Ref	Ref	Ref	Ref
RAVC	28.6 (21.7–35.4)	269	136	160	2.7% (1.8–3.3)	254	75	$1,472	$2,670	$1,248
Human and mosquito reservoir[#]										
RACD only	52.5 (30.4–74.6)	238	Ref	Ref	3.1% (2.0–4.1)	140	Ref	Ref	Ref	Ref
rfMDA+RAVC	24.6 (14.7–34.5)	111	127	150	1.8% (0.5–2.9)	81	59	$842	$1,812	$714

RACD: Reactive case detection, rfMDA: Reactive focal Mass Drug administration, RAVC: Reactive focal vector control, ICER: Incremental cost effectiveness ratio, DALY: disability-adjusted life year.

[#]Based on half of the clusters (14)

For peer review only - http://bmjopen.bmj.com/site/about/guidelines.xhtml

Table 4. Results of sensitivity analysis and scenario analyses (cost in 2017 USD)

Parameter	Base value/ Sensitivity analysis value(s)	rfMDA Costs per PAR	rfMDA ICER (*vs* RACD) per incident case averted	RAVC Costs per PAR	RAVC ICER (*vs* No RAVC) per incident case averted	rfMDA+RAVC Costs per PAR	rfMDA+RAVC ICER (*vs* RACD only) per incident case averted
Reference	Predicted incidence*, Discount rate 3%, Personnel salaries on partner scale, Actellic 300 CS cost $38.80	$41.4	$114	$48.8	$1,472	$54.4	$842
Intervention effectiveness*	Lower 95% CI	NA	$63	NA	$996	NA	$625
	Upper 95% CI	NA	$679	NA	$2,781	NA	$1,304
Discount rate	0%	$41.2	$109	$48.6	$1,464	$54.1	$840
	5%	$41.5	$116	$48.9	$1,477	$54.5	$851
Personnel	Government scale	$32.0	$86.1	$37.9	$1,145	$42.2	$659
	CHW	$35.2	$110	$40.4	$1,290	$45.2	$743
Actellic 300 CS	$15	NA	NA	$47.5	$1,381	$52.9	$799

Costs per PAR are NA for intervention effectiveness because cost inputs did not change.

Note that for personnel costs, government scale refers to use of the government pay scale for all staff. CHW refers to use of the CHW pay scale for nurses, data collectors, and spray operators. Other positions (drivers and office staff) utilized a partner pay scale.

*95% CIs for predicted incidences (per 1000 person-years) are: 19–40 (rfMDA), 21.7–35.4 (RAVC), and 14.7–34.5 (rfMDA+RAVC)

RACD: reactive case detection, rfMDA: reactive focal mass drug administration, RAVC: reactive focal vector control, ICER: Incremental cost effectiveness ratio, CHW: community health worker, NA: not applicable.

Table 5. Personnel time (Minutes)

	rfMDA	RACD	RAVC
Personnel-minutes per participant enrolled (median, IQR)	30 (25–38)	38 (35–44)	NA
Personnel-minutes per structure sprayed (median, IQR)	NA	NA	34 (29–39)
Personnel minutes per individual protected with RAVC (median, IQR)	NA	NA	9 (7–12)
Preparation time (Minutes) (median, IQR)	33 (27–39)	32 (26–38)	22.5 (17–28.5)
Travel time to and from community (Minutes)	124 (112–137)	126 (113–140)	138 (122–149)

For peer review only - http://bmjopen.bmj.com/site/about/guidelines.xhtml

Figure legends

Figure 1. Cost category breakdown for RACD, rfMDA, RAVC

Figure 2. Start-up and recurrent costs by comparison groups for cost per event (2a), cost per person (2b), and cost per population at risk (2c).

Figure 3. Incremental cost effectiveness ratio (ICER) of RACD *vs* rfMDA, no RAVC *vs* RAVC and RACD only *vs* rfMDA+RAVC per incident case averted, prevalent infection averted, and DALY averted.

Figure 4. Tornado diagram of change in the incremental cost effectiveness ratio per incident case averted for rfMDA *vs* RACD (4a), RAVC *vs* No RAVC (4b) and rfMDA+RAVC *vs* RACD only (4c). The values in brackets are the range over which the parameter was varied. The vertical line is the baseline value of the ICER per incident case averted. The blue bars show the direction and magnitude of change in the ICER, when the input variable is set to its lower value and the red bars show the direction and magnitude of change when the input variable is set to its higher value. Marginal changes in the ICER are not visible on the graph.

For peer review only

For peer review only - http://bmjopen.bmj.com/site/about/guidelines.xhtml

Declarations

Ethical approval and consent to participate

The main trial received ethical approval from the Namibia Ministry of Health and Social Services, and the Institutional Review Boards of the University of Namibia, University of California San Francisco and London School of Medicine and Hygiene. Consent was not obtained from study staff being observed as it was considered part of study supervision.

Consent for publication

All authors have read and approved the final manuscript.

Data availability statement

Data supporting the conclusions of this article is available upon request.

Competing Interests

The authors have no competing interests to report.

Funding

This work was supported by grants from the Novartis Foundation (A122666), the Bill and Melinda Gates Foundation (OPP1138299), and Horchow Family Fund, (grant 5300375400 to MSH).

Authors' contributions

MSH, RG, and IK led the conception and study design. DM, JS, AB, HS, and PU contributed to the study design. HN led the data collection with support from CSG, VS, BZ, MT, LMP, LW, LS and AM. HN and MSH performed data cleaning and data analyses. JN, BW and MSD supported data analysis. HN wrote the first draft of the manuscript. HN and MSH wrote the final draft of the manuscript. All authors read and approved the final manuscript.

For peer review only
For peer review only - http://bmjopen.bmj.com/site/about/guidelines.xhtml

Acknowledgements

The authors would like to express their gratitude to the national and regional Ministry of Health and Social Services, and the local traditional leadership for their support. The authors would also like to acknowledge Megan McElroy, Simataa Nyathi, Flavian Libita, Chaze Sibeya, and Katie Fox for their support in the preparation and collection of field data.

For peer review only

References

1. Feachem RG, Phillips AA, Hwang J, et al. Shrinking the malaria map: progress and prospects. *Lancet*. 2010;376(9752):1566–1578. doi:10.1016/S0140-6736(10)61270-6: 10.1016/S0140-6736(10)61270-6

2. WHO. *World Malaria Report 2019*.; 2019.

3. Okell LC, Ghani AC, Lyons E, Drakeley CJ. Submicroscopic Infection in Plasmodium falciparum-Endemic Populations: A Systematic Review and Meta-Analysis. *J Infect Dis*. 2009;200(10):1509-1517. doi:10.1086/644781

4. Sturrock HJW, Hsiang MS, Cohen JM, et al. Targeting Asymptomatic Malaria Infections : Active Surveillance in Control and Elimination. *PLoS Med*. 2013;10(6):e1001467. doi:10.1371/journal.pmed.1001467

5. Björkman A, Cook J, Sturrock H, et al. Spatial Distribution of Falciparum Malaria Infections in Zanzibar : Implications for Focal Drug Administration Strategies Targeting Asymptomatic Parasite Carriers. *Clin Infect Dis*. 2017;64(9):1236-1243. doi:10.1093/cid/cix136

6. Bousema T, Okell L, Felger I, Drakeley C. Asymptomatic malaria infections: detectability, transmissibility and public health relevance. *Nat Rev Microbiol*. 2014;12(12):833-840.

7. Moonen B, Cohen JM, Tatem AJ, et al. A framework for assessing the feasibility of malaria elimination. *Malar J*. 2010;9(1):322. doi:10.1186/1475-2875-9-322

8. Cotter C, Sturrock HJW, Hsiang MS, et al. The changing epidemiology of malaria elimination : new strategies for new challenges. *Lancet*. 2013;382(9895):900-911. doi:10.1016/S0140-6736(13)60310-4

9. Stresman GH, Baidjoe AY, Stevenson J, et al. Focal Screening to Identify the Subpatent Parasite Reservoir in an Area of Low and Heterogeneous Transmission in the Kenya Highlands. *J Infect Dis*. 2015;212:1768-1777. doi:10.1093/infdis/jiv302

10. Cook J, Xu W, Msellem M, et al. Mass Screening and Treatment on the Basis of Results of a Plasmodium falciparum -Speci fi c Rapid Diagnostic Test Did Not Reduce Malaria Incidence in Zanzibar. *J Infect Dis*. 2015;211:1476-1483. doi:10.1093/infdis/jiu655

11. WHO. *Evidence Review Group Report on Mass Drug Administration , Mass Screening and Treatment and Focal Screening and Treatment for Malaria*.; 2015.

12. Smith JL, Auala J, Tambo M, et al. Spatial clustering of patent and sub-patent malaria infections in northern Namibia: Implications for surveillance and response strategies for elimination. *PLoS One*. 2017;12(8):e0180845. doi:10.1371/journal.pone.0180845

13. Hsiang MS, Ntuku H, Roberts KW, et al. Effectiveness of reactive focal mass drug administration and reactive focal vector control to reduce malaria transmission in the low malaria-endemic setting of Namibia: a cluster-randomised controlled, open-label, two-by-two factorial design trial. *Lancet*. 2020;395(10233):1361-1373. doi:10.1016/S0140-6736(20)30470-0

14. Zelman BW, Baral R, Zarlinda I, et al. Costs and cost ‐ effectiveness of malaria reactive case detection using loop ‐ mediated isothermal amplification compared to microscopy in the low transmission setting of Aceh Province , Indonesia. *Malar J*. Published online 2018:1-12.

For peer review only - http://bmjopen.bmj.com/site/about/guidelines.xhtml

doi:10.1186/s12936-018-2361-y

15. Larson BA, Ngoma T, Silumbe K, et al. A framework for evaluating the costs of malaria elimination interventions : an application to reactive case detection in Southern Province of Zambia , 2014. *Malar J*. 2016;15(408):1-15. doi:10.1186/s12936-016-1457-5

16. Yukich JO, Scott C, Silumbe K, et al. Cost-Effectiveness of Focal Mass Drug Administration and Mass Drug Administration with Dihydroartemisinin–Piperaquine for Malaria Prevention in Southern Province, Zambia: Results of a Community-Randomized Controlled Trial. *Am J Trop Med Hyg*. 2020;103(2_Suppl):46-53. doi:10.4269/ajtmh.19-0661

17. Kaneko A, Taleo G, Kalkoa M, Yamar S, Kobayakawa T, Björkman A. Malalria eradictaion on islands. *Lancet*. 2000;356.

18. Bath D, Cook J, Govere J, et al. Effectiveness and cost-effectiveness of reactive, targeted indoor residual spraying for malaria control in low-transmission settings: a cluster-randomised, non-inferiority trial in South Africa. *Lancet (London, England)*. 2021;397(10276):816-827. doi:10.1016/S0140-6736(21)00251-8

19. Ministry of Health and Social Services. Namibia. *National Policy on Malaria*.; 2014.

20. Medzihradsky O, Kleinschmidt I, Mumbengegwi D, Roberts K, McCreesh P, Dufour K. Study protocol for a cluster randomised controlled factorial design trial to assess the effectiveness and feasibility of reactive focal mass drug administration and vector control to reduce malaria transmission in the low endemic setting of Namibia. *BMJ Open*. 2018;8(e019294).

21. World Health Organization. *Indoor Residual Spraying: An Operational Manual for Indoor Residual Spraying (IRS) for Malaria Transmission Control and Elimination.*

22. Drummond M, Sculper M, Torrnace G, O'Brien B, Stoddart G. *Methods for the Economic Evaluation of Health Care Programmes*. 3rd ed. (Oxford, ed.). Oxford University Press; 2005.

23. OANDA. Currency converter. Accessed October 9, 2019. http://www.oanda.com/currency/historical-rates/

24. Walker D, Kumaranayake L. Allowing for differential timing in cost analyses : discounting and annualization. *Health Policy Plan*. 2002;17(1):112-118.

25. Lopetegui M, Yen P-Y, Lai A, Jeffries J, Embi P, Payne P. Time motion studies in healthcare: what are we talking about? *J Biomed Inform*. 2014;49:292-299. doi:10.1016/j.jbi.2014.02.017

26. Murray CJL, Vos T, Lozano R, et al. Disability-adjusted life years (DALYs) for 291 diseases and injuries in 21 regions, 1990-2010: a systematic analysis for the Global Burden of Disease Study 2010. *Lancet (London, England)*. 2012;380(9859):2197-2223. doi:10.1016/S0140-6736(12)61689-4

27. WHO. Global Health Observatory Data. Accessed May 20, 2015. http//www.who.int/gho/mortality_burden_disease/life_tables/en

28. Gunda R, Chimbari MJ, Mukaratirwa S. Assessment of Burden of Malaria in Gwanda District, Zimbabwe, Using the Disability Adjusted Life Years. *Int J Environ Res Public Health*. 2016;13(2):244. doi:10.3390/ijerph13020244

29. World Bank. International Comparison Program database. Published 2017.

For peer review only - http://bmjopen.bmj.com/site/about/guidelines.xhtml

www.data.worldbank.org/indicator/

30. WHO. Cost effectiveness and strategic planning (WHO-CHOICE). http://www.who.int/choice/cost-effectiveness/en/

31. Sabot O, Cohen JM, Hsiang MS, et al. Costs and financial feasibility of malaria elimination. *Lancet*. 2010;376(9752):1604-1615. doi:10.1016/S0140-6736(10)61355-4

32. Shretta R, Avanceña AL V, Hatefi A. The economics of malaria control and elimination: a systematic review. *Malar J*. 2016;15(1):593. doi:10.1186/s12936-016-1635-5

33. White MT, Conteh L, Cibulskis R, Ghani AC. Costs and cost-effectiveness of malaria control interventions - a systematic review. *Malar J*. 2011;10(1):337. doi:10.1186/1475-2875-10-337

34. Zelman BW, Baral R, Zarlinda I, et al. Costs and cost - effectiveness of malaria reactive case detection using loop - mediated isothermal amplification compared to microscopy in the low transmission setting of Aceh Province , Indonesia. *Malar J*. Published online 2018:1-12. doi:10.1186/s12936-018-2361-y

35. Silumbe K, Yukich JO, Hamainza B, et al. Costs and cost-effectiveness of a large-scale mass testing and treatment intervention for malaria in. *Malar J*. 2015;14(211):1-13. doi:10.1186/s12936-015-0722-3

36. Drake TL, Okello G, Njagi K, et al. Cost analysis of school-based intermittent screening and treatment of malaria in Kenya. *Malar J*. 2011;10(1):273. doi:10.1186/1475-2875-10-273

37. Vaughan K, Kok MC, Witter S, Dieleman M. Costs and cost-effectiveness of community health workers : evidence from a literature review. *Hum Resour Health*. 2015;13:71. doi:10.1186/s12960-015-0070-y

38. Liverani M, Nguon C, Sok R, et al. Improving access to health care amongst vulnerable populations: a qualitative study of village malaria workers in Kampot, Cambodia. *BMC Health Serv Res*. 2017;17(1):335. doi:10.1186/s12913-017-2282-4

39. Eisele TP, Bennett A, Silumbe K, et al. Impact of Four Rounds of Mass Drug Administration with Dihydroartemisinin–Piperaquine Implemented in Southern Province, Zambia. *Am J Trop Med Hyg*. 2020;103(2_Suppl):7-18. doi:https://doi.org/10.4269/ajtmh.19-0659

40. Okebe J, Ribera JM, Balen J, et al. Reactive community-based self- administered treatment against residual malaria transmission : study protocol for a randomized controlled trial. *Trials*. Published online 2018:1-8.

41. Galactionova K, Velarde M, Silumbe K, et al. Costing malaria interventions from pilots to elimination programmes. *Malar J*. 2020;19(1):332. doi:10.1186/s12936-020-03405-3

42. Antillon M, Huang C-I, Rock KS, Tediosi F. Economic evaluation of disease elimination: An extension to the net-benefit framework and application to human African trypanosomiasis. *Proc Natl Acad Sci U S A*. 2021;118(50). doi:10.1073/pnas.2026797118

For peer review only - http://bmjopen.bmj.com/site/about/guidelines.xhtml

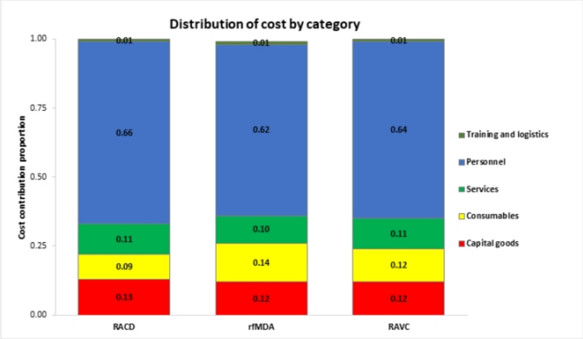

Figure 1. Cost category breakdown for RACD, rfMDA, RAVC

338x190mm (96 x 96 DPI)

a)

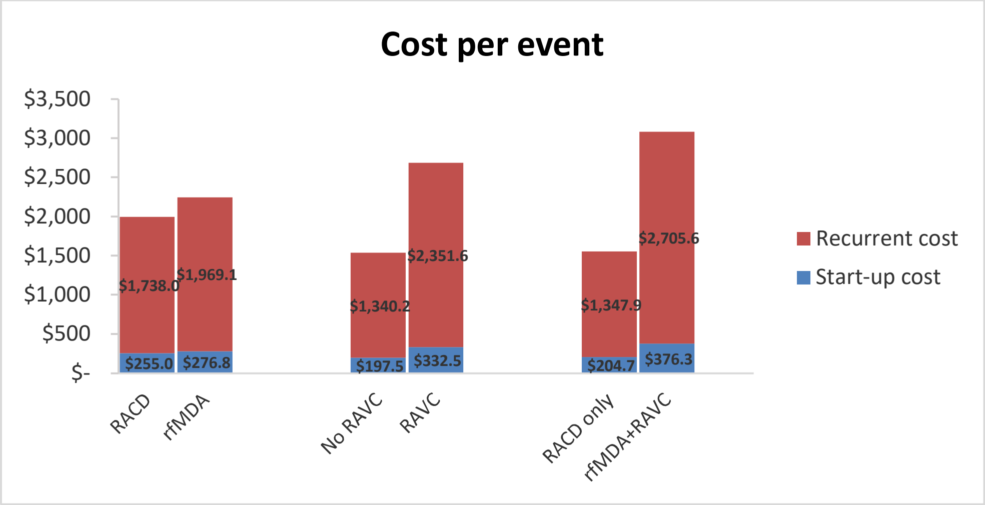
Cost per event
$3,500
$3,000
$2,500
$2,000
$1,500
$1,000
$500
$-
$1,738.0
$255.0
$1,969.1
$276.8
$1,340.2
$197.5
$2,351.6
$332.5
$1,347.9
$204.7
$2,705.6
$376.3
RACD
rfMDA
No RAVC
RAVC
RACD only
rfMDA+RAVC
Recurrent cost
Start-up cost

b)

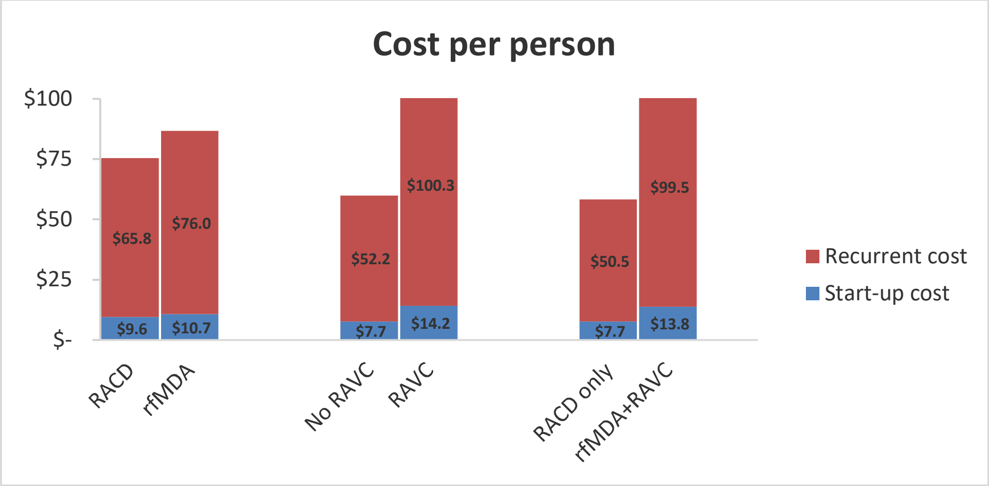
Cost per person
$100
$75
$50
$25
$-
$65.8
$9.6
$76.0
$10.7
$52.2
$7.7
$100.3
$14.2
$50.5
$7.7
$99.5
$13.8
RACD
rfMDA
No RAVC
RAVC
RACD only
rfMDA+RAVC
Recurrent cost
Start-up cost

c)

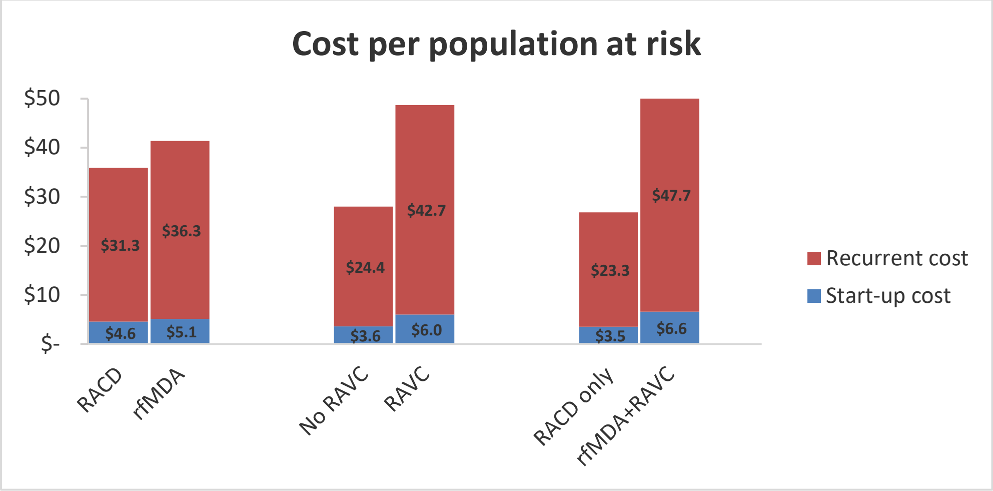
Cost per population at risk
$50
$40
$30
$20
$10
$-
$31.3
$4.6
$36.3
$5.1
$24.4
$3.6
$42.7
$6.0
$23.3
$3.5
$47.7
$6.6
RACD
rfMDA
No RAVC
RAVC
RACD only
rfMDA+RAVC
Recurrent cost
Start-up cost

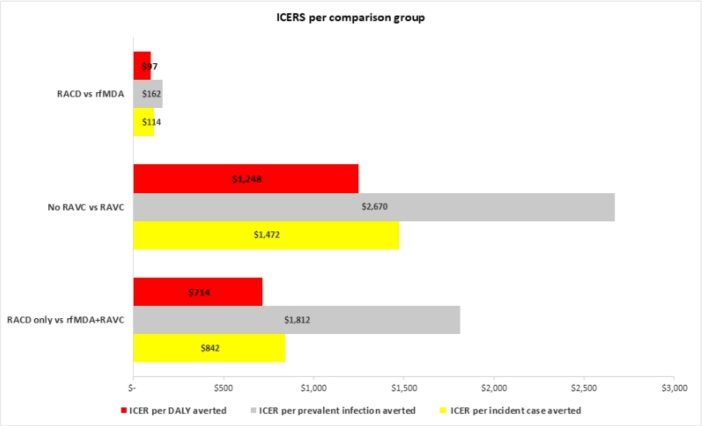

Figure 3. Incremental cost effectiveness ratio (ICER) of RACD vs rfMDA, no RAVC vs RAVC and RACD only vs rfMDA+RAVC per incident case averted, prevalent infection averted, and DALY averted.

338x190mm (96 x 96 DPI)

a)

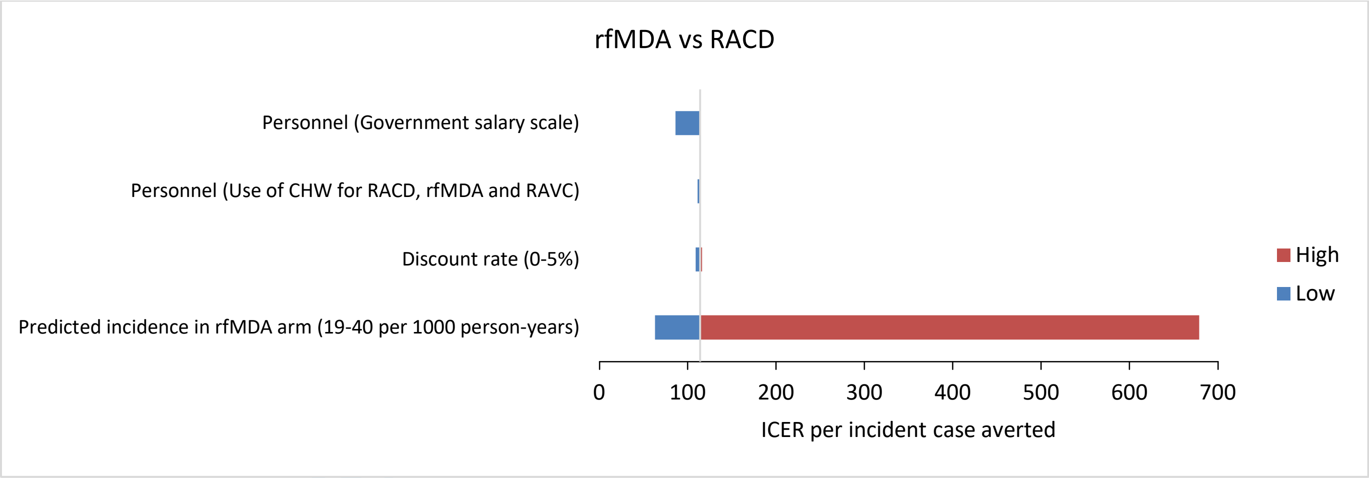
rfMDA vs RACD
Personnel (Government salary scale)
Personnel (Use of CHW for RACD, rfMDA and RAVC)
Discount rate (0-5%)
Predicted incidence in rfMDA arm (19-40 per 1000 person-years)
High
Low
0
100
200
300
400
500
600
700
ICER per incident case averted

b)

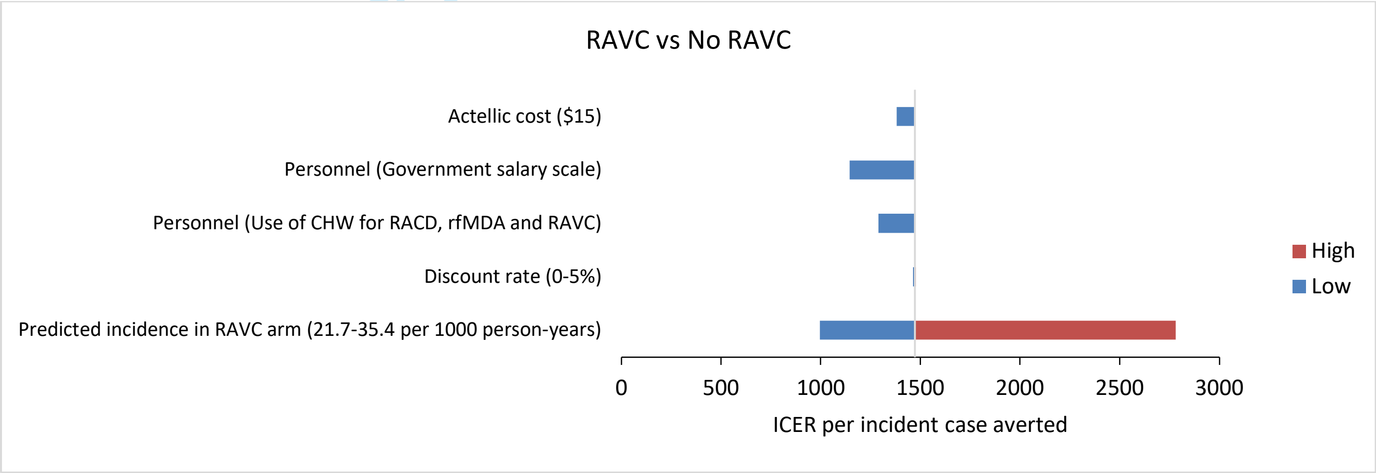
RAVC vs No RAVC
Actellic cost ($15)
Personnel (Government salary scale)
Personnel (Use of CHW for RACD, rfMDA and RAVC)
Discount rate (0-5%)
Predicted incidence in RAVC arm (21.7-35.4 per 1000 person-years)
High
Low
0
500
1000
1500
2000
2500
3000
ICER per incident case averted

c)

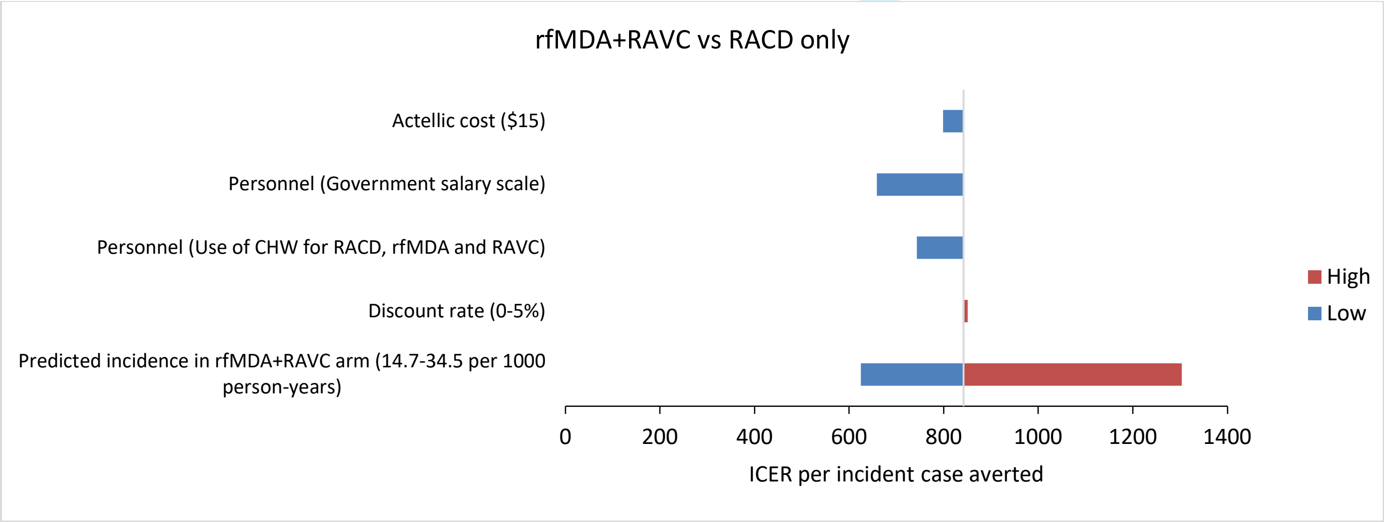
rfMDA+RAVC vs RACD only
Actellic cost ($15)
Personnel (Government salary scale)
Personnel (Use of CHW for RACD, rfMDA and RAVC)
Discount rate (0-5%)
Predicted incidence in rfMDA+RAVC arm (14.7-34.5 per 1000 person-years)
High
Low
0
200
400
600
800
1000
1200
1400
ICER per incident case averted

Supplement to:

Cost and cost-effectiveness of reactive case detection (RACD), reactive focal mass drug administration (rfMDA), and reactive focal vector control (RAVC) to reduce malaria in the low endemic setting of Namibia: an analysis alongside a 2x2 factorial design cluster randomized controlled trial

Table of Contents

Appendix 1a. Cumulative incidence of locally acquired malaria 2
Appendix 1b. Prevalence of quantitative PCR-detected infection 3
Appendix 2. Comparison groups 4
Appendix 3. Namibia Government salary scale 5

For peer review only

Appendix 1a. Cumulative incidence of locally acquired malaria

	No. of EAs	Incidence per 1000 person-years (95% CI)	p value	Incidence rate ratio (95% CI)	p value	Adjusted incidence rate ratio (95% CI)	p value
**Human reservoir**							
RACD	27	38.3 (23.0–53.6)	0.26	1 (reference)	0.51	1 (reference)	0.009
rfMDA	28	30.8 (12.8–48.7)		0.82 (0.26–1.37)		0.52 (0.16–0.88)	
**Mosquito reservoir**							
No RAVC	27	38.9 (20.7–57.1)	0.23	1 (reference)	0.41	1 (reference)	0.002
RAVC	28	30.2 (15.0–45.5)		0.78 (0.26–1.30)		0.48 (0.16–0.80)	
**Mosquito and human reservoir**							
RACD only	13	41.4 (21.5–61.2)	0.11	1 (reference)	0.32	1 (reference)	0.006
rfMDA+RAVC	14	25.0 (5.2–44.7)		0.62 (0.24–1.59)		0.26 (0.10–0.68)	

RACD: reactive case detection, rfMDA: reactive focal mass drug administration, RAVC: reactive vector control

For peer review only

Appendix 1b. Prevalence of quantitative PCR-detected infection

	N	Prevalence (95% CI)	p value	Prevalence ratio (95% CI)	p value	Adjusted prevalence ratio (95% CI)	p value
**Human reservoir**							
RACD	2150	3.78% (2.85-5.00)	0.46	1 (reference)	0.92	1 (reference)	0.009
rfMDA	1932	3.16% (2.14-4.65)		1.05 (0.03-2.07)		0.59 (0.21-0.98)	
**Mosquito reservoir**							
No RAVC	2030	4.07% (2.92-5.64)	0.15	1 (reference)	0.13	1 (reference)	0.002
RAVC	2052	2.92% (2.13-3.99)		0.61 (0.10-1.12)		0.36 (0.13-0.59)	
**Human and mosquito reservoir**							
RACD only	1016	3.70% (2.39-5.69)	0.04	1 (reference)	0.17	1 (reference)	0.004
rfMDA+RAVC	918	1.75% (0.99-3.09)		0.52 (0.20-1.32)		0.16 (0.05-0.55)	

RACD: reactive case detection, rfMDA: reactive focal mass drug administration, RAVC: reactive vector control

For peer review only

Appendix 2. Comparison groups

**rfMDA *versus* RACD**			Human reservoir	
			**RACD (28 clusters)**	**rfMDA (28 clusters)**
	Mosquito reservoir	**No RAVC**	RACD only	rfMDA only
		**RAVC**	RACD + RAVC	rfMDA + RAVC

**RAVC *versus* No RAVC**			Human reservoir	
			**RACD**	**rfMDA**
	Mosquito reservoir	**No RAVC (28 clusters)**	RACD only	rfMDA only
		**RAVC (28 clusters)**	RACD + RAVC	rfMDA + RAVC

**rfMDA +RAVC *versus* RACD only**			Human reservoir	
			**RACD**	**rfMDA**
	Mosquito reservoir	**No RAVC**	RACD only **(14 clusters)**	rfMDA only
		**RAVC**	RACD + RAVC	rfMDA + RAVC **(14 clusters)**

RACD: Reactive case detection, RAVC: Reactive vector control
rfMDA: Reactive focal Mass Drug administration

For peer review only

Appendix 3. Namibia Government salary scale

Grade	Low per annum		High per annum	
15	NAD	35,777	NAD	46,521
14	NAD	54,682	NAD	71,105
13	NAD	72,556	NAD	94,349
12	NAD	99,633	NAD	119,501
11	NAD	122,965	NAD	147,485
10	NAD	147,485	NAD	176,895
9	NAD	180,505	NAD	216,499
8	NAD	220,828	NAD	263,911
7	NAD	269,189	NAD	321,707
6	NAD	328,139	NAD	392,158
5	NAD	400,001	NAD	478,220
4	NAD	478,220	NAD	502,753
3	NAD	512,809	NAD	544,196
2	NAD	555,080	NAD	589,055
1	NAD	600,837	NAD	637,612
1C	NAD	625,110	NAD	663,373
1B	NAD	650,365	NAD	690,172
1A	NAD	663,373	NAD	703,975

For peer review only

CHEERS checklist—Items to include when reporting economic evaluations of health interventions

Section/item	Item No	Recommendation	Reported on page No/ line No
**Title and abstract**			
Title	1	Identify the study as an economic evaluation or use more specific terms such as "cost-effectiveness analysis", and describe the interventions compared.	p. 1, line 1
Abstract	2	Provide a structured summary of objectives, perspective, setting, methods (including study design and inputs), results (including base case and uncertainty analyses), and conclusions.	p. 2, all lines
**Introduction**			
Background and objectives	3	Provide an explicit statement of the broader context for the study.	p. 3-4, all lines
		Present the study question and its relevance for health policy or practice decisions.	p. 4, line 21-24; p. 5, line 1-4
**Methods**			
Target population and subgroups	4	Describe characteristics of the base case population and subgroups analysed, including why they were chosen.	p. 5, line 8 to end; p. 6, line 1-3
Setting and location	5	State relevant aspects of the system(s) in which the decision(s) need(s) to be made.	p. 5, line 8-15
Study perspective	6	Describe the perspective of the study and relate this to the costs being evaluated.	p. 5, line 8 to end; p. 6, line 1-3
Comparators	7	Describe the interventions or strategies being compared and state why they were chosen.	p. 4, line 21-24, p. 5, line 1-3
Time horizon	8	State the time horizon(s) over which costs and consequences are being evaluated and say why appropriate.	p. 5, line 8
Discount rate	9	Report the choice of discount rate(s) used for costs and	p. 7, line 8-11

For peer review only

Section/item	Item No	Recommendation	Reported on page No/ line No
		outcomes and say why appropriate.	
Choice of health outcomes	10	Describe what outcomes were used as the measure(s) of benefit in the evaluation and their relevance for the type of analysis performed.	p. 9, line 3<br>p. 5, line 18-19<br>p. 6, 14-19
Measurement of effectiveness	11a	*Single study-based estimates:* Describe fully the design features of the single effectiveness study and why the single study was a sufficient source of clinical effectiveness data.	p. 5, line 7-end<br>p. 6, 1-13
	11b	*Synthesis-based estimates:* Describe fully the methods used for identification of included studies and synthesis of clinical effectiveness data.	n/a
Measurement and valuation of preference based outcomes	12	If applicable, describe the population and methods used to elicit preferences for outcomes.	p. 5, line 7-20
Estimating resources and costs	13a	*Single study-based economic evaluation:* Describe approaches used to estimate resource use associated with the alternative interventions. Describe primary or secondary research methods for valuing each resource item in terms of its unit cost. Describe any adjustments made to approximate to opportunity costs.	p. 6, line 20-p8, line 12
	13b	*Model-based economic evaluation:* Describe approaches and data sources used to estimate resource use associated with model health states. Describe primary or secondary research methods for valuing each resource item in terms of its unit cost. Describe any adjustments made to approximate to opportunity costs.	n/a
Currency, price date, and conversion	14	Report the dates of the estimated resource quantities and unit costs.	p. 7, line 1-4

For peer review only

Section/item	Item No	Recommendation	Reported on page No/ line No
		Describe methods for adjusting estimated unit costs to the year of reported costs if necessary. Describe methods for converting costs into a common currency base and the exchange rate.	
Choice of model	15	Describe and give reasons for the specific type of decision-analytical model used. Providing a figure to show model structure is strongly recommended.	p. 9, line 3 to p. 10, line 8
Assumptions	16	Describe all structural or other assumptions underpinning the decision-analytical model.	p. 9, line 22 to p. 10, line 12
Analytical methods	17	Describe all analytical methods supporting the evaluation. This could include methods for dealing with skewed, missing, or censored data; extrapolation methods; methods for pooling data; approaches to validate or make adjustments (such as half cycle corrections) to a model; and methods for handling population heterogeneity and uncertainty.	p. 9, line 22 to p. 11, line 2.
**Results**			
Study parameters	18	Report the values, ranges, references, and, if used, probability distributions for all parameters. Report reasons or sources for distributions used to represent uncertainty where appropriate. Providing a table to show the input values is strongly recommended.	p. 11, line 9 to p. 13, line 4
Incremental costs and outcomes	19	For each intervention, report mean values for the main categories of estimated costs and outcomes of interest, as well as mean differences between the comparator groups. If applicable, report incremental cost-effectiveness ratios.	p. 11, line 9<br>p. 12, line 12

For peer review only

Section/item	Item No	Recommendation	Reported on page No/ line No
Characterising uncertainty	20a	*Single study-based economic evaluation:* Describe the effects of sampling uncertainty for the estimated incremental cost and incremental effectiveness parameters, together with the impact of methodological assumptions (such as discount rate, study perspective).	p. 12, line 14 to 19
	20b	*Model-based economic evaluation:* Describe the effects on the results of uncertainty for all input parameters, and uncertainty related to the structure of the model and assumptions.	n/a
Characterising heterogeneity	21	If applicable, report differences in costs, outcomes, or cost-effectiveness that can be explained by variations between subgroups of patients with different baseline characteristics or other observed variability in effects that are not reducible by more information.	p. 12, line 14 to 19
**Discussion**			
Study findings, limitations, generalisability, and current knowledge	22	Summarise key study findings and describe how they support the conclusions reached. Discuss limitations and the generalisability of the findings and how the findings fit with current knowledge.	p. 13, line 6 to p. 16, line 8
**Other**			
Source of funding	23	Describe how the study was funded and the role of the funder in the identification, design, conduct, and reporting of the analysis. Describe other non-monetary sources of support.	p. 22, line 14-15
Conflicts of interest	24	Describe any potential for conflict of interest of study contributors in accordance with journal policy. In the absence of a journal policy, we recommend authors comply with	p. 22, line 12

For peer review only

Section/item	Item No	Recommendation	Reported on page No/ line No
		International Committee of Medical Journal Editors recommendations.	

For consistency, the CHEERS statement checklist format is based on the format of the CONSORT statement checklist

For peer review only
